# Study of blood flow patterns in a stenosed artery through the combined effect of body acceleration and generalized womersley solution

**DOI:** 10.1038/s41598-025-85566-2

**Published:** 2025-01-13

**Authors:** Mahesh C. Udupa, Sunanda Saha, Sekarapandian Natarajan

**Affiliations:** 1 Department of Mathematics, School of Advanced Sciences, VIT, Vellore, 632014 India; 2 Centre for Clean Environment, VIT, Vellore, 632014 India; 3 Computational Fluid Dynamics Laboratory, School of Mechanical Engineering, VIT, Vellore, 632014 India

**Keywords:** Mathematics and computing, Applied mathematics

## Abstract

Stenosis causes the narrowing of arteries due to plaque buildup, which impedes blood flow and affects flow dynamics. This work numerically analyzes flow fluctuations in stenosed arteries under realistic physiological conditions (resting and exercise) and external body acceleration. The artery is inclined at angle $\Theta$, and blood rheology is modeled using a generalized power-law fluid. A modified two-dimensional SIMPLE pressure-correction-based numerical solver with orthogonal coordinate transformation simulates blood flow. A generalized Womersley solution is imposed at the inlet. We validate the solver and perform simulations to assess the influence of geometric and flow parameters, analyzing time-averaged and phase-averaged data. We investigate the correlation between hyperviscosity and physiological conditions, finding that exercise increases recirculation downstream of the stenosis. We also study the impact of transitioning between resting and exercise conditions, noting that the transition rate correlates with stenosis development, indicating potential complications.

## Introduction

The circulatory system’s primary function is to circulate blood throughout the body to deliver oxygen to various body parts^1^. Atherosclerosis is one type of arterial blood flow obstruction that might have detrimental effects^2^. Atherosclerosis is a condition where cholesterol builds up in the intima and underlying smooth muscle of an artery, leading to plaque (stenosis) that causes blockages, reduces compliance, and impacts pressure and resistance in the arterial system^3^. Several studies^4–6^offer detailed insights into the pathogenesis of atherosclerosis. The pulsatile quality of arterial blood flow, which is the outcome of the heart’s regular pumping motion, is an important characteristic. An additional factor that can complicate blood flow dynamics is hyperviscosity^7^, which has become notably linked to COVID-19 in recent times^8–10^. Hyperviscosity is a condition where the viscosity of blood exceeds its normal range, usually greater than 0.005 Pa$\cdot$s^11^. This condition forces the heart to work harder to pump blood, potentially resulting in premature death^12^, even in younger individuals. Hyperviscosity hampers blood flow in the blood vessels^13^and heightens the likelihood of forming blood clots^14^. Therefore, studying local blood flow in arteries using numerical simulations and mathematical modeling can help us better understand arterial stenosis and make it easier to develop innovative treatments that lower mortality. The degree of stenosis, which is determined by the percentage decrease in the artery lumen, and the geometry of the stenosis are the two primary factors influencing blood flow. This work focuses on the latter aspect.

The literature has several significant analytical investigations on blood flow in arterial stenosis. Using pulsatility and non-Newtonian qualities as examples, Chaturani and Samy^15^ investigated the effects on flow dynamics. Using Reynolds number as a perturbation parameter, Ponalagusamy et al.^16^examined blood flow with various stenosis geometries. Sriyab^17^examined the blood flow in narrow arteries with mild bell-shaped stenosis, treating blood as a non-Newtonian fluid and employing the K-L model. In one of the following works, Sriyab^18^ conducted a comparative examination of the same in arteries with cosine-shaped and bell-shaped stenosis. Utilizing a non-Newtonian power law model, they determined that the blood flow in bell-shaped geometry results in greater skin friction than that in cosine-shaped geometry. Awan et al.^19^investigated the impacts of body acceleration on blood flow in an elliptical-shaped stenotic artery at various positions along its wall, especially when the artery is inclined with respect to the horizontal. The study treated blood as a Casson fluid and observed that both body acceleration and the inclination angle increase the flow rate. Notably, the mentioned analytical studies do not account for pressure variations in the radial direction and, more importantly, exclude the critical inertial terms from the momentum equations. Srivastava^20^analyzed blood flow patterns in a porous inclined stenotic artery under the influence of the magnetic field. Owasit and Sruyab^21^ compared flow characteristics in bell-shaped and cosine-shaped stenoses under vertical symmetry and asymmetry conditions. The study revealed distinct and significant differences in the flow behavior between the two shapes of stenosis. Hussain et al.^22^ have studied blood flow behavior under stenosis suppositions with the influence of nanoparticles. Shahzad et al.^23^ examined non-Newtonian blood flow in a multi-stenosed, elliptically-shaped artery using the Carreau fluid model. The results indicated that non-Newtonian effects dominated along the radial axis. However, the mentioned studies do not account for the unsteadiness in blood flow and assume the flow to be steady.

The current literature includes research examining how the shape of stenosis affects overall blood flow dynamics, with particular emphasis on the stress applied to plaques. The literature has several significant analytical investigations on blood flow in arterial stenosis. Using pulsatility and non-Newtonian qualities as examples, Chaturani and Samy^15^investigated the effects on flow dynamics. In a related study, Ponalagusamy and Selvi^24^ took into account a non-zero slip velocity at the artery wall. Using the Reynolds number as a perturbation parameter, Ponalagusamy et al.^16^ examined blood flow with various stenosis geometries. Using OpenFOAM, Ozden et al.^25^studied the effect of six different shapes on flow fluctuations, specifically in the post-stenotic region, where they found that superimposition of more than one stenosis increases vortical activity. Furthermore, by defining the slopes at the entrance and exit of elliptical stenosis using a shape parameter, a number of researchers examined the impact of the distance between the two points. These investigations often conclude that lowering the entrance slope reduces flow resistance as well as wall shear stress^26–28^. However, the mentioned works do not consider the incompressible non-Newtonian blood flow subjected to a generalized Womersley inlet flow condition under the influence of external body acceleration. External body accelerations like those experienced during travel, gym workouts, running, and sports activities impact human life. These accelerations can lead to health issues such as increased pulse rate and headaches. Researchers have also investigated the effects of body acceleration on pulsatile flow through stenotic arteries. Nagarani and Sarojamma^29^explored how external body acceleration influences Casson fluid flow in an artery with a circular cross-section and mild stenosis. Their findings revealed an increase in flow rate under body acceleration. In their modeling of a two-dimensional, non-Newtonian blood flow in a stenosed artery under the impact of body acceleration, Haghighi^30^discovered a direct relationship between the amplitudes of body acceleration and flow rate. Numerous other studies have investigated the influence of external body acceleration on blood flow in constricted arteries^31–33^. However, there is no report in the numerical literature addressing the impact of body acceleration coupled with both physiological resting and exercise conditions.

The governing equations for fluid flow might differ throughout numerical investigations; these often employ the primitive variables approach or the vorticity-stream function formulation. Under the primitive variable formulations, many of the proposed numerical models^15,34–40^ in the literature reduce the radial momentum equation to $\frac {\partial p}{\partial r}=0$ assuming that the pressure waves’ wavelength is noticeably greater than the lumen radius. However, some of the other studies in the literature^41–45^do not make this assumption and retain the governing equations in both axial and radial directions. Many popular pressure correction techniques that are used to solve governing equations in the form of primitive variables include methods such as Simultaneous Variable Adjustment (SIVA)^46^, Marker and Cell approach (MAC)^47,48^, Semi-Implicit Method for Pressure Linked Equations (SIMPLE)^49–51^, and Pressure Implicit with Splitting of Operator (PISO)^52,53^. Some of the CFD works^54,55^also incorporate fluid-structure interaction (FSI) to stimulate tissue degradation and its effects on blood flow. A few other works incorporating one of the above methods to perform numerical simulation using CFD software include^56–60^. However, the discussion on the application of the analytically derived generalized Womersley inlet condition under the influence of body acceleration on non-Newtonian blood flow has not yet been reported in the literature. Further, Udupa et al.^61^ have investigated the influence of generalized Womersley solution at the inlet of a stenosed artery on the pressure drop and consequential physical parameters. However, the work accounted for the Newtonian model, which in the current work we are extending with a non-Newtonian model in addition to the objectives as stated below.

In the current work, we develop a two-dimensional pressure-correction-based numerical solver to simulate non-Newtonian blood flow in a stenosed artery, for which, we consider, (*i*) Orthogonal coordinate transformation to convert the curved domain onto a rectangular one, (*ii*) Generalised Womersley solution^62^ at the inlet, (*iii*) effect of external body acceleration. A comprehensive derivation of the solver is presented in the manuscript. Furthermore, we analyze the influence of variation in shapes of stenosis and other geometrical parameters on flow fluctuations. Finally, we study the correlation between hyperviscosity and the effects of body acceleration during both resting and exercise conditions and its consequent flow field patterns in a periodic cardiac cycle, which, to the best of the authors’ knowledge, is not reported in the numerical literature. The whole analysis is structured by dividing the influence of geometric and flow parameters on the time-averaged and phase-averaged flow fields.

The remainder of this paper is as follows: Section 1 focuses on the definitions and the detailed schematics of the geometries considered with their relevance. Section 2 introduces the governing equations and the initial and boundary conditions, with the corresponding coordinate transformation, to enhance the computational efficiency. Section 3 discusses in detail the derivation of the numerical discretization of the governing equations in the transformed coordinates. Section 4 presents the results of our parametric studies, beginning with a validation of the considered solver, followed by the influence of geometrical parameters alone on the flow field, and then the influence of flow parameters alone on the flow field. Section 5 provides a conclusion and the future scope of this work.

## Geometrical models

**Fig. 1:**
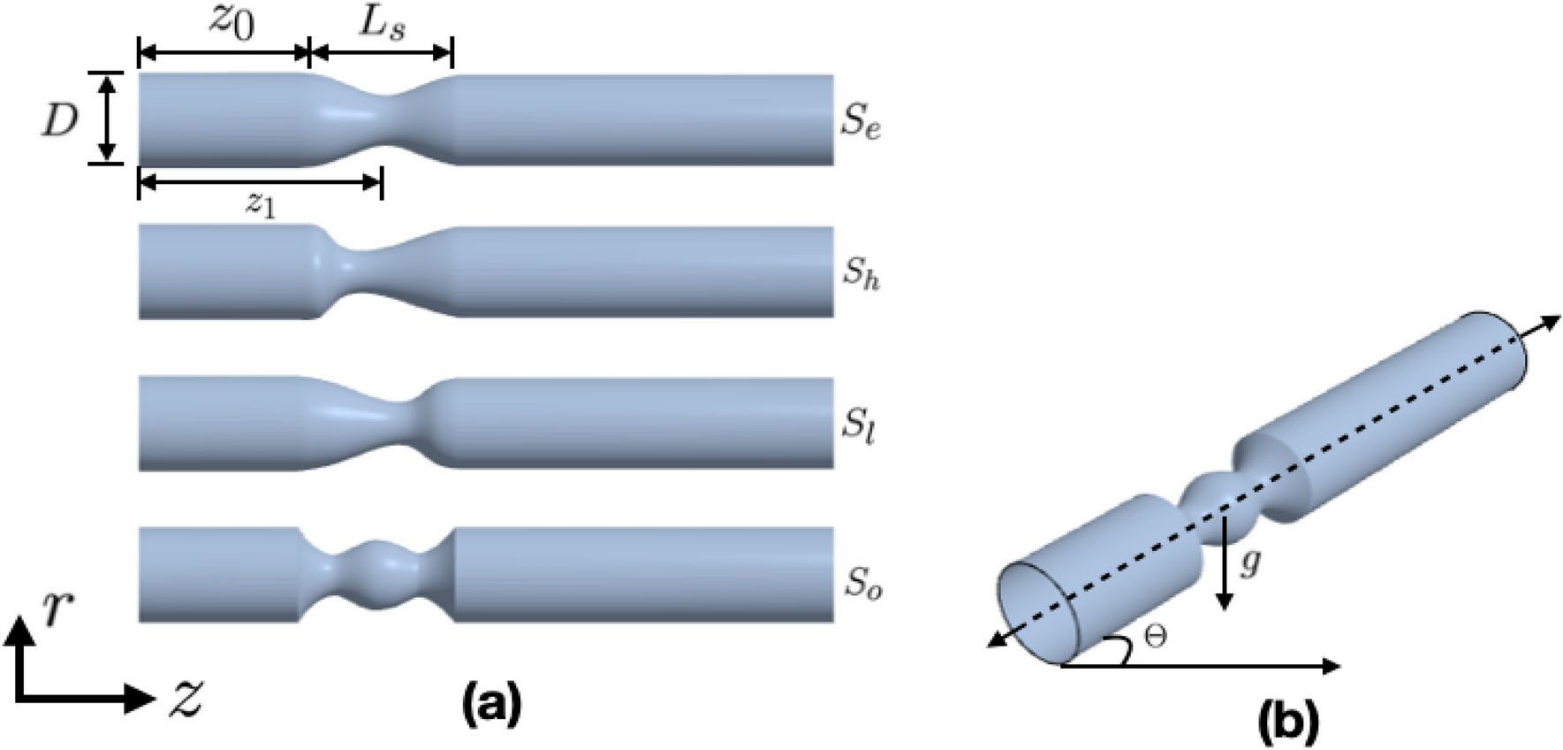
Schematic of the two-dimensional Geometrical models. The three-dimensional viewpoint is for visualization purposes only. (**a**) Different shapes of stenosis, (**b**) Arterial domain inclined at an angle $\Theta$ with the horizontal.

Various shapes of vascular stenosis are reported in the medical literature^63–65^. Four idealized models of stenosed vessels have been derived by analyzing these images along with similar ones. In Fig. 1, we provide a schematic illustration of the domain considered for the numerical simulation, showcasing the geometric characteristics of stenotic arteries. Each one is predicated on a healthy internal carotid artery of radius, $r_{0}$. For all models, $L_{s}$ is the length of the stenosis, *D* is the diameter of the non-stenosed vessel, $z_{1}$ is the axial distance of the center of the stenosis from the inlet, $z_{0}$ is the separation between the inlet and the beginning of the stenosis.

The arterial radius, *R*(*z*), is defined below for each shape.

1. Elliptical shaped stenosis ($S_{e}$): Commonly considered in many of the numerical studies^66^, elliptical-shaped stenosis (Fig. 1(a)) can be considered as the base case here, and it is defined as,
  1 $$\begin{aligned} R(z) = {\left\{ \begin{array}{ll} r_{0}\left[ 1 - \dfrac{\alpha }{2} \left( 1 + \cos \left( \dfrac{ \pi (z - z_{1})}{ z_{0}} \right) \right) \right] , \hspace{11mm} z_{1} - {\dfrac{L_{s}}{2}} \le z \le z_{1} + {\dfrac{L_{s}}{2} }, \\ r_{0}, \hspace{64mm} \text{ otherwise }, \end{array}\right. } \end{aligned}$$
  where $\alpha$ correlates to the severity of the stenosis.
2. High Slope ($S_{h}$) and Low Slope shaped stenosis ($S_{l}$): High and low slope models (Fig. 1(a)) are employed to understand the significance of the distance between the stenosis throat and its exit. The radii are defined^27^ as,
  2 $$\begin{aligned} & R(z) = {\left\{ \begin{array}{ll} r_{0}\left( 1 - A_{s} \left( L_{s}^{(m-1)}(z-z_{0}) - (z-z_{0})^{m} \right) \right) , \hspace{11mm} z_{1} - {\dfrac{L_{s}}{2}} \le z \le z_{1} + {\dfrac{L_{s}}{2} }, \\ r_{0}, \hspace{86mm} \text{ otherwise }, \end{array}\right. } \nonumber \\ & \text{ and, } \hspace{5mm} A_{s} = \dfrac{2 \delta _{s}}{DL_{s}^{m}} \dfrac{m^{\frac {m}{(m-1)}}}{(m-1)}, \end{aligned}$$
  where the high and low slope models are denoted by values of 1.5 and 6.0, respectively, for the shape parameter *m*. The measurement of $\delta _{s}$ at the stenosis throat determines the distance between the vessel wall and the stenosis throat.
3. Overlapping shaped stenosis ($S_{o}$): The investigation of the presence of double constrictions and the resulting irregularities is conducted using the overlapping model (Fig. 1(a)), defined^67^ as
  3 $$\begin{aligned} R(z) = {\left\{ \begin{array}{ll} r_{0} \left( 1 - \dfrac{3 \delta _{s}}{DL_{s}^{4}} \left( 11(z-z_{0})L_{s}^{3} - 47(z-z_{0})^{2} L_{s}^{2} \right. \right. \\ \left. \left. \hspace{25mm} + 72(z-z_{0})^{3} L_{s} - 36(z-z_{0})^{4}\right) \right) , \hspace{20mm} z_{1} - {\dfrac{L_{s}}{2}} \le z \le z_{1} + {\dfrac{L_{s}}{2} }, \\ r_{0}, \hspace{100mm} \text{ otherwise }. \end{array}\right. } \end{aligned}$$

In the remainder of the paper, for brevity, we refer to the four cases (Fig. 1(a)) discussed above as Shape ($S_{j}$), where $j\in \{e,h,l,o\}$.

## Governing equations for the fluid

We study the incompressible, two-dimensional, unstable laminar blood flow through a stenotic-arterial section. Our focus is on the one-way coupling of blood flow, as indicated in Li et al.^68^, meaning that the pressure gradient does not influence the compliance of the wall. Furthermore, we consider that the flow is axisymmetric ($\frac {\partial }{\partial \theta } = 0$). As a result, the mass and momentum equations in the cylindrical coordinates have the following dimensional conservative form^69^,

4 $$\begin{aligned} & \dfrac{1}{\bar{r}} \dfrac{\partial }{\partial \bar{r}}(\bar{r}\bar{u}) + \dfrac{\partial \bar{w}}{\partial \bar{z}} = 0, \end{aligned}$$

5 $$\begin{aligned} & \rho \left[ \dfrac{\partial \bar{w}}{\partial \bar{t}} + \dfrac{\partial (\bar{u}\bar{w})}{\partial \bar{r}} + \dfrac{\partial \bar{w}^{2}}{\partial \bar{z}} + \dfrac{\bar{u}\bar{w}}{\bar{r}}\right] = - \dfrac{\partial \bar{p}}{\partial \bar{z}} + \left[ \dfrac{1}{\bar{r}} \dfrac{\partial }{\partial \bar{r}} \left( \bar{r} \bar{\tau }_{rz} \right) + \dfrac{\partial }{\partial \bar{z}} (\bar{\tau }_{zz}) \right] + \rho g \sin {\Theta } + G(t), \end{aligned}$$

6 $$\begin{aligned} & \rho \left[ \dfrac{\partial \bar{u}}{\partial \bar{t}} + \dfrac{\partial (\bar{u}\bar{w})}{\partial \bar{z}} + \dfrac{\partial \bar{u}^{2}}{\partial \bar{r}} + \dfrac{\bar{u}^{2}}{\bar{r}}\right] = - \dfrac{\partial \bar{p}}{\partial \bar{r}} + \left[ \dfrac{1}{\bar{r}} \dfrac{\partial }{\partial \bar{r}} \left( \bar{r} \bar{\tau }_{rr}\right) + \dfrac{\partial }{\partial \bar{z}} (\bar{\tau }_{rz}) \right] + \rho g \cos {\Theta }, \end{aligned}$$

where $\bar{p}$ represents the pressure in $\text{ kgm}^{-1}\text{ s}^{-2}$, $\rho$ represents the density in $\text{ kgm}^{-3}$, and $\bar{u}$ and $\bar{w}$ are the radial and axial velocities in $\text{ ms}^{-1}$, respectively, $\bar{\tau }$ is stress tensor in $\text{ kgm}^{-1}\text{ s}^{-2}$, *g* is the acceleration due to gravity in $\text{ ms}^{-2}$, and $\Theta$ is the angle of inclination (Fig. 1(b)) in radians. *G*(*t*) represents the body acceleration term given by $G(t) = a_{0} \cos (\omega _{b} t - \phi )$; $a_{0}$ is amplitude with units $\text{ kgm}^{-2}\text{ s}^{-2}$, $\omega _{b} = 2 \pi f_{b}$, $f_{b}$ is the frequency in $\text{ s}^{-1}$, and $\phi$ is the phase angle in radians.

7 $$\begin{aligned} \bar{\tau }_{rr}= & 2 \bar{\mu }_{p} \left( \dfrac{\partial \bar{u}}{\partial \bar{r}}\right) , \hspace{10mm} \bar{\tau }_{rz} = \bar{\mu }_{p} \left( \dfrac{\partial \bar{u}}{\partial \bar{z}} + \dfrac{\partial \bar{w}}{\partial \bar{r}} \right) , \hspace{10mm} \bar{\tau }_{zz} = 2 \bar{\mu }_{p} \left( \dfrac{\partial \bar{w}}{\partial \bar{z}} \right) , \end{aligned}$$

8 $$\begin{aligned} \bar{\mu }_{p}= & K \left( |\bar{\dot{\nu }}|\right) ^{{\frac {\left( n-1\right) )}{2}}}, \end{aligned}$$

9 $$\begin{aligned} |\bar{\dot{\nu }}|= & 2\left[ \left( \dfrac{\partial \bar{u}}{\partial \bar{r}} \right) ^2 + \left( \dfrac{\bar{u}}{\bar{r}}\right) ^2 + \left( \dfrac{\partial \bar{w}}{\partial \bar{z}} \right) ^2 \right] + \left( \dfrac{\partial \bar{u}}{\partial \bar{z}} + \dfrac{\partial \bar{w}}{\partial \bar{r}}\right) ^{2}, \end{aligned}$$

where K (units: Pa $\text{ s}^{n}$ $==$ kg $\text{ m}^{-1}$ $\text{ s}^{n-2}$) is flow consistence index and *n* (dimensionless) is flow behaviour index. The following parameters are used to express the non-dimensional version of Eqs. (4)-(6),

10 $$\begin{aligned} r = \dfrac{\bar{r}}{r_{0}}, \hspace{2mm} z = \dfrac{\bar{z}}{r_{0}}, \hspace{2mm} u = \dfrac{\bar{u}}{u_{0}}, \hspace{2mm} w = \dfrac{\bar{w}}{u_{0}}, \hspace{2mm} t = \dfrac{\bar{t} u_{0}}{r_{0}}, \hspace{2mm} p = \dfrac{\bar{p}}{\rho u_{0}^{2}}, \hspace{2mm} Re = \dfrac{\rho u_{0}^{2-n} r_{0}^{n}}{K}, \hspace{2mm} Fr = \dfrac{u_{0}}{\sqrt{g r_{0}}}, \hspace{2mm} \Lambda = \dfrac{a_{0}}{P_{0}}. \end{aligned}$$

where $r_{0}$ is the characteristic length that corresponds to the artery’s radius, $u_{0}$ is the average cross-sectional velocity of the fluid, *Re* is the Reynolds number, *Fr* is the Froude’s number, and $\Lambda$ is a constant that decides the amplitude of the body acceleration. Using Eq. (10), we write the non-dimensional form of the governing equations as follows

11 $$\begin{aligned} & \left[ \dfrac{1}{r} \dfrac{\partial }{\partial r}(ru) + \dfrac{\partial w}{\partial z} \right] = 0, \end{aligned}$$

12 $$\begin{aligned} & \left[ \dfrac{\partial w}{\partial t} + \dfrac{\partial (uw)}{\partial r} + \dfrac{\partial w^{2}}{\partial z} + \dfrac{uw}{r}\right] = - \dfrac{\partial p}{\partial z} \nonumber \\ & \quad + \dfrac{1}{Re} \left[ \left( \dfrac{\gamma }{r} \left\{ \dfrac{\partial u}{\partial z} + \dfrac{\partial w}{\partial r} \right\} \right) + \left( \gamma \dfrac{\partial }{\partial r} \left\{ \dfrac{\partial u}{\partial z} + \dfrac{\partial w}{\partial r} \right\} \right) + \left\{ \dfrac{\partial u}{\partial z} + \dfrac{\partial w}{\partial r} \right\} \dfrac{\partial \gamma }{\partial r} + 2 \gamma \dfrac{\partial ^{2} w}{\partial z^{2}} + 2 \dfrac{\partial w}{\partial z} \dfrac{\partial \gamma }{\partial z} \right] \nonumber \\ & \quad + \dfrac{\sin {\Theta }}{{Fr}^{2}} + \dfrac{4 \Lambda }{Re} \cos ({\omega _{b}t + \phi }), \end{aligned}$$

13 $$\begin{aligned} & \left[ \dfrac{\partial u}{\partial t} + \dfrac{\partial (uw)}{\partial z} + \dfrac{\partial u^{2}}{\partial r} + \dfrac{u^{2}}{r}\right] = - \dfrac{\partial p}{\partial r} \nonumber \\ & \quad + \dfrac{1}{Re} \left[ \left( \dfrac{2 \gamma }{r} \dfrac{\partial u}{\partial r} \right) + 2 \gamma \dfrac{\partial ^{2} u}{\partial r^{2}} + 2 \dfrac{\partial u}{\partial r} \dfrac{\partial \gamma }{\partial r} + \gamma \dfrac{\partial }{\partial z} \left( \dfrac{\partial u}{\partial z} + \dfrac{\partial w}{\partial r} \right) + \left( \dfrac{\partial u}{\partial z} + \dfrac{\partial w}{\partial r} \right) \dfrac{\partial \gamma }{\partial z} \right] + \dfrac{\cos {\Theta }}{{Fr}^{2}}. \end{aligned}$$

where,

$$\begin{aligned} \gamma = \{\dot{\nu }\}^{\frac {(n-1)}{2}}; \hspace{10mm} \{\dot{\nu }\}= & \left\{ 2\left[ \left( \dfrac{\partial u}{\partial r} \right) ^2 + \left( \dfrac{u}{r}\right) ^2 + \left( \dfrac{\partial w}{\partial z} \right) ^2 \right] + \left( \dfrac{\partial u}{\partial z} + \dfrac{\partial w}{\partial r}\right) ^{2} \right\} . \end{aligned}$$

### Initial and boundary conditions

For the unknowns *u*, *w*, and *p* in Eqs. (11)-(13), the initial condition is provided by

14 $$\begin{aligned} & u(r,z,0) = 0, \hspace{3mm} w(r,z,0) = 0, \hspace{3mm} p(r,z,0) = 0, \hspace{15mm} \text{ at } \hspace{1mm} t = 0. \end{aligned}$$

The unknowns found in Eqs. (11)-(13) have the following boundary conditions,

15 $$\begin{aligned} & u(0,z,t) = 0, \hspace{3mm} \dfrac{\partial w(0,z,t)}{\partial r} = 0, \hspace{3mm} \dfrac{\partial p(0,z,t)}{\partial r} = 0, \hspace{9.0mm} \text{ about } \text{ the } \text{ line } \text{ of } \text{ symmetry } (r = 0), \end{aligned}$$

16 $$\begin{aligned} & u(R,z,t) = 0, \hspace{3mm} w(R,z,t) = 0, \hspace{4mm} \dfrac{\partial p(R,z,t)}{\partial r} = 0, \hspace{9mm} \text{ at } \text{ the } \text{ wall } (r = R(z)). \end{aligned}$$

Non-reflective boundary conditions are imposed at the outlet ($z = 2d$), given by

17 $$\begin{aligned} \dfrac{\partial u(r,2d,t)}{\partial z} = 0, \hspace{3mm} \dfrac{\partial w(r,2d,t)}{\partial z} = 0. \end{aligned}$$

We apply a generalized Womersley solution at the inlet ($z = 0$), which consists of a pulsatile component dependent on (*r*, *t*) paired with a parabolic velocity profile. For the same, the mathematical expression^62^ is given by,

18 $$\begin{aligned} w(r,0,t) \hspace{0mm}= & \hspace{0mm} 2\left[ 1-\left( \frac{r}{R}\right) ^{2} \right] - 2 \sum ^{\infty }_{n=1} e^{\left( -v_{n}^{2}/Re \right) t} \left[ \frac{J_{0}(v_{n}) - J_{0}\left( \frac{r v_{n}}{R}\right) }{v_{n}J_{1}(v_{n})} \right] \nonumber \\ & + \frac{i}{2} \left[ \frac{J_{0}(v_{a}) - J_{0}\left( \frac{r v_{a}}{R}\right) }{J_{2}(v_{a})} e^{2 \pi \frac {f_{i}r_{0}}{u_{0}}} - \frac{J_{0}(v_{b}) - J_{0}\left( \frac{r v_{b}}{R}\right) }{J_{2}(v_{b})} e^{- 2 \pi \frac {f_{i}r_{0}}{u_{0}}} \right] \nonumber \\ & + \sum ^{\infty }_{n=1} e^{\left( -v_{n}^{2}/Re \right) t} \frac{{W\hspace{-.7mm}o}^{2}}{{W\hspace{-.7mm}o}^{4} + v_{n}^{4}} \left[ \frac{2 v_{n}\left( J_{0}(v_{n}) - J_{0}\left( \dfrac{r v_{n}}{R}\right) \right) }{J_{1}(v_{n})}\right],\end{aligned}$$

where $v_{a} = \sqrt{- i} {W\hspace{-.7mm}o}$, $v_{b} = \sqrt{i} {W\hspace{-.7mm}o}$, ${{W\hspace{-.7mm}o}} = \sqrt{\dfrac{\rho \omega r_{0}^{2}}{\mu }}$, represents Womersley number, $\omega$ = 2$\pi f_{i}$, where $f_{i}$ is the velocity inlet pulsatile frequency, The Bessel functions of first kind of order 0 and 1 are denoted by $J_{0}$ and $J_{1}$, respectively, while the $n^{th}$ zero of the Bessel’s function of the second kind is represented by $v_{n}$.

### Coordinate transformation

To reduce computational complexity, we carry out a radial coordinate transformation, expressed as $x = \frac {r}{R(z)}$, to convert the physical domain into a rectangular computational space. The modified form of Eqs. (11)-(13) following the application of the coordinate transformation is as follows,

19 $$\begin{aligned} & \dfrac{\partial (x u)}{\partial x} + xR \dfrac{\partial w}{\partial z} - x^{2} \dfrac{\partial w}{\partial x} \dfrac{\partial R}{\partial z} = 0, \end{aligned}$$

20 $$\begin{aligned} & \dfrac{\partial w}{\partial t} = \dfrac{x}{R} \dfrac{\partial R}{\partial t} \dfrac{\partial w}{\partial x} - \dfrac{1}{R} \dfrac{\partial (uw)}{\partial x} - \dfrac{\partial w^{2}}{\partial z} + A \dfrac{\partial w^{2}}{\partial x} - \dfrac{uw}{xR} - \dfrac{\partial p}{\partial z} + A \dfrac{\partial p}{\partial x} \nonumber \\ & \hspace{7mm} + \dfrac{1}{Re} \left[ \dfrac{\gamma }{xR} \left\{ \dfrac{\partial u}{\partial z} - A \dfrac{\partial u}{\partial x} + \dfrac{1}{R} \dfrac{\partial w}{\partial x}\right\} + \left( \dfrac{\gamma }{R} \left\{ \dfrac{\partial ^{2} u}{\partial {z}\partial x} - A \dfrac{\partial ^{2} u}{\partial x^{2}} - \dfrac{1}{R} \dfrac{\partial R}{\partial z} \dfrac{\partial u}{\partial x} + \dfrac{1}{R} \dfrac{\partial ^{2} w}{\partial x^{2}} \right\} \right) \right. \nonumber \\ & \left. \hspace{7mm} + \dfrac{1}{R} \left( \left\{ \dfrac{\partial u}{\partial z} - A \dfrac{\partial u}{\partial x} + \dfrac{1}{R} \dfrac{\partial w}{\partial x}\right\} \dfrac{\partial \gamma }{\partial x} \right) \right. \nonumber \\ & \left. \hspace{7mm} + 2 \gamma \left( \dfrac{\partial ^{2} w}{\partial z^{2}} - 2 A \dfrac{\partial ^{2} w}{\partial {z} \partial {x}} - \dfrac{x}{R} \dfrac{\partial ^{2}R}{\partial z^{2}} \dfrac{\partial w}{\partial x} + A^{2} \dfrac{\partial ^{2} w}{\partial x^{2}} \right) + 2 \left( \dfrac{\partial w}{\partial z} - A \dfrac{\partial w}{\partial x}\right) \left( \dfrac{\partial \gamma }{\partial z} - A \dfrac{\partial \gamma }{\partial x}\right) \right] \nonumber \\ & \hspace{8mm}+ \dfrac{\sin {\Theta }}{{Fr}^{2}} + \dfrac{4 \Lambda }{Re} \cos ({\omega _{b}t + \phi }), \end{aligned}$$

21 $$\begin{aligned} & \dfrac{\partial u}{\partial t} = \dfrac{x}{R} \dfrac{\partial R}{\partial t} \dfrac{\partial u}{\partial x} - \dfrac{1}{R} \dfrac{\partial (u^{2})}{\partial x} - \dfrac{\partial uw}{\partial z} + A \dfrac{\partial uw}{\partial x} - \dfrac{u^{2}}{xR} - \dfrac{1}{R}\dfrac{\partial p}{\partial x} \nonumber \\ & \hspace{7mm} + \dfrac{1}{Re} \left[ \left( \dfrac{2 \gamma }{xR^{2}} \dfrac{\partial u}{\partial x}\right) + \dfrac{2 \gamma }{R^{2}} \dfrac{\partial ^{2} u}{\partial x^{2}} + \dfrac{2}{R^{2}} \dfrac{\partial u}{\partial x} \dfrac{\partial \gamma }{\partial x} \right. \nonumber \\ & \left. \hspace{7mm} + \gamma \left( \dfrac{\partial ^{2} u}{\partial {z}^{2} } - A \dfrac{\partial ^{2} u}{\partial {z} \partial {x}} + \dfrac{x}{R^{2}} \left( \dfrac{\partial R}{\partial z}\right) \dfrac{\partial u}{\partial x} - \dfrac{x}{R} \dfrac{\partial ^{2} R}{\partial {z}^{2}} \dfrac{\partial u}{\partial x} + \dfrac{1}{R} \dfrac{\partial ^{2} w}{\partial {z} \partial {x}} - \dfrac{1}{R^{2}} \dfrac{\partial R}{\partial z} \dfrac{\partial w}{\partial x} \right) \right. \nonumber \\ & \left. \hspace{7mm} + \gamma A \left( \dfrac{\partial ^{2} u}{\partial {z} \partial {x}} - A \dfrac{\partial ^{2}u}{\partial x^{2}} - \dfrac{1}{R} \dfrac{\partial R}{\partial z} \dfrac{\partial u}{\partial x} + \dfrac{1}{R} \dfrac{\partial ^{2} w}{\partial x^{2}}\right) + \left( \dfrac{\partial u}{\partial z} - A \dfrac{\partial u}{\partial x} + \dfrac{1}{R} \dfrac{\partial w}{\partial x} \right) \left( \dfrac{\partial \gamma }{\partial z} - A \dfrac{\partial \gamma }{\partial x}\right) \right] \nonumber \\ & \hspace{7mm} + \dfrac{\cos {\Theta }}{{Fr}^{2}}, \end{aligned}$$

where,

$$\begin{aligned} A &= \frac{x}{R}\frac{\partial R}{\partial z}, \\ \gamma= & \{\dot{\nu }\}^{\frac {(n-1)}{2}} = \left\{ 2 \left[ \left( \frac{1}{R}\frac{\partial u}{\partial x} \right) ^{2} + \left( \frac{u}{xR}\right) ^{2} + \left( \frac{\partial w}{\partial z} - A \frac{\partial w}{\partial x}\right) ^{2} \right] + \left( \frac{\partial u}{\partial z} - A \frac{\partial u}{\partial x} + \frac{1}{R} \frac{\partial w}{\partial x}\right) ^{2} \right\} ^{\frac {(n-1)}{2}}, \\ & \frac{\partial \gamma }{\partial z} = \frac{(n-1)}{2} \gamma ^{\frac {(n-3)}{2}} \left\{ 4 \left[ \left( \frac{1}{R} \frac{\partial u}{\partial x}\right) \left( \frac{1}{R} \frac{\partial ^{2} u}{\partial {z} \partial x} - \frac{1}{R^{2}} \frac{\partial R}{\partial z} \frac{\partial u}{\partial x}\right) + \left( \frac{u}{xR} \right) \left( xR \frac{\partial u}{\partial z} - xu \frac{\partial R}{\partial z} \right) \left( \frac{1}{(xR)^2}\right) \right. \right. \\ & \left. \left. + \left( \frac{\partial w}{\partial z} - A \frac{\partial w}{\partial x}\right) \left( \frac{\partial ^{2} w}{\partial z^{2}} - A \frac{\partial ^{2}w}{\partial {z} \partial x} - \frac{x}{R} \frac{\partial ^{2} R}{\partial z^{2}} \frac{\partial w}{\partial x} + \frac{x}{R^{2}} \left( \frac{\partial R}{\partial z}\right) ^2 \frac{\partial w}{\partial x} \right) \right] \right. \\ & \left. + 2 \left( \frac{\partial u}{\partial z} - A \frac{\partial u}{\partial x} + \frac{1}{R} \frac{\partial w}{\partial x}\right) \left( \frac{\partial ^{2} u}{\partial z^{2}} - A \frac{\partial ^{2}u}{\partial {z} \partial x} - \frac{x}{R} \frac{\partial ^{2} R}{\partial z^{2}} \frac{\partial u}{\partial x} + \frac{x}{R^{2}} \left( \frac{\partial R}{\partial z}\right) ^2 \frac{\partial u}{\partial x} - \frac{1}{R}\frac{\partial ^{2} w}{\partial z^{2}} + \frac{1}{R^{2}} \frac{\partial w}{\partial z} \right) \right\} ,\\ & \frac{\partial \gamma }{\partial x} = \frac{(n-1)}{2} \gamma ^{\frac {(n-3)}{2}} \left\{ 4 \left[ \left( \frac{1}{R} \frac{\partial u}{\partial x}\right) \left( \frac{1}{R} \frac{\partial ^{2} u}{\partial x^{2}}\right) + \left( \frac{u}{xR} \right) \left( xR \frac{\partial u}{\partial x} - R u \right) \frac{1}{(xR)^{2}} + \left( \frac{\partial w}{\partial z} - A \frac{\partial w}{\partial x}\right) \right. \right. \\ & \left. \left. \left( \frac{\partial ^{2} w}{\partial {z} \partial {x}} - A \frac{\partial ^{2} w}{\partial x^{2}} - \frac{1}{R} \frac{\partial R}{\partial z} \frac{\partial w}{\partial x} \right) \right] + 2 \left( \frac{\partial u}{\partial z} - A \frac{\partial u}{\partial x} + \frac{1}{R} \frac{\partial w}{\partial x}\right) \left( \frac{\partial ^{2} u}{\partial {z} \partial {x}} - A \frac{\partial ^{2} u}{\partial x^{2}} - \frac{1}{R} \frac{\partial R}{\partial z} \frac{\partial u}{\partial x} + \frac{1}{R} \frac{\partial ^{2} w}{\partial x^{2}} \right) \right\}. \end{aligned}$$

Similarly, the modified form of the initial and boundary conditions (Eqs. (14)-(18)) takes on the following form once the coordinate transformation is applied,

22 $$\begin{aligned} & u(x,z,0) = 0, \hspace{4mm} w(x,z,0) = 0, \hspace{4mm} p(x,z,0) = 0, \hspace{16mm} \text{ at } \hspace{1mm} t = 0, \end{aligned}$$

23 $$\begin{aligned} & u(0,z,t) = \dfrac{\partial w(0,z,t)}{\partial x} = \dfrac{\partial p(0,z,t)}{\partial x} = 0, \hspace{27mm} \text{ at } \hspace{1mm} x = 0, \end{aligned}$$

24 $$\begin{aligned} & u(1,z,t) = w(1,z,t) = \dfrac{\partial p(1,z,t)}{\partial x} = 0, \hspace{23mm} \text{ at } \hspace{1mm} x = 1, \end{aligned}$$

25 $$\begin{aligned} & \dfrac{\partial u(x,2d,t)}{\partial z} = 0, \hspace{3mm} \dfrac{\partial w(x,2d,t)}{\partial z} = 0, \hspace{34mm} \text{ at } \hspace{1mm} z = 2d, \end{aligned}$$

26 $$\begin{aligned} & w(x,0,t) \hspace{2mm} = \hspace{2mm} 2\left[ 1-x^{2} \right] - 2 \sum ^{\infty }_{n=1} e^{\left( -v_{n}^{2}/Re \right) t} \left[ \frac{J_{0}(v_{n}) - J_{0}\left( {x v_{n}}\right) }{v_{n}J_{1}(v_{n})} \right] \nonumber \\ & \hspace{20mm}+ \frac{i}{2} \left[ \dfrac{J_{0}(v_{a}) - J_{0}\left( {x v_{a}}\right) }{J_{2}(v_{a})} e^{2 \pi \frac {f_{i}r_{0}}{u_{0}}} - \dfrac{J_{0}(v_{b}) - J_{0}\left( {x v_{b}}\right) }{J_{2}(v_{b})} e^{- 2 \pi \frac {f_{i}r_{0}}{u_{0}}} \right] \nonumber \\ & \hspace{20mm} + \sum ^{\infty }_{n=1} e^{\left( -v_{n}^{2}/Re \right) t} \frac{{W\hspace{-.7mm}o}^{2}}{{W\hspace{-.7mm}o}^{4} + v_{n}^{4}} \left[ \dfrac{2 v_{n}\left( J_{0}(v_{n}) - J_{0}\left( {x v_{n}}\right) \right) }{J_{1}(v_{n})}\right] . \end{aligned}$$

## Numerical discretization and solution algorithm

We employ the finite difference method to numerically solve Eqs (19)-(21) on a staggered Cartesian grid. The initial and boundary conditions are applied as described in Eqs. (22)-(26). It is important to preserve mass conservation for the velocity field at each infinitesimal point during the time evolution of the solution while simulating incompressible flow. Strong coupling between the continuity and momentum equations is necessary to meet this physical limitation, with the pressure field serving as a Lagrange multiplier. As a result, the velocity field can reliably satisfy the continuity requirement. Since there is no explicit evolution equation for pressure in Eqs. (19)-(21), solving the coupled system and simultaneously computing velocity and pressure values at each time step becomes challenging. To expedite the procedure and develop a productive solver for modeling incompressible blood flow in a stenotic artery, we utilize the SIMPLE correction-based approach. The governing equations (19)-(21) are discretized on a staggered grid, the details of which are given in Appendix A, provided in supplementary.

The cell centers and faces of a staggered cell^61^ are oriented perpendicularly to both the axial and radial directions. Using $x=i\Delta {x}$ and $z=j\Delta {z}$, where *i*, *j* are the discrete indices and $\Delta {x}$, $\Delta {z}$ are the grid spacing for *x* and *z* coordinates, we represent a grid position on the *xz* plane. Time instants are obtained by $t = k\Delta {t}$, where *k* is the index and $\Delta {t}$ is the time step. The radial and axial coordinates of any cell face are found as follows: $z_{lj}$ = $z_{j}$ + $\frac {\Delta {z}}{2}$; $x_{li}$ = $x_{i}$ + $\frac {\Delta {x}}{2}$. The coordinates of the cell center preceding the face under examination are denoted by $x_{i}$ and $z_{j}$ in this instance. For a more detailed discussion on the structure of the staggered grid and the schemes considered, we refer the reader to the work of Udupa et al.^61^.

The unknowns in Eqs. (A1)-(A3) are $w^{k+1}_{i,lj}$, $u^{k+1}_{li,j}$, and $p^{k+1}_{i,j}$. Due to the absence of an evolution equation for pressure, determining both the pressure field and the velocity field simultaneously at the $(k+1)$th time level poses a challenge. The goal is to satisfy the continuity constraint described in Eq. (A1), ensuring that the divergence of the velocity field remains zero. In order to do this, we compute the velocity and pressure fields while maintaining the continuity condition using a pressure-correction-based technique. Using this method, we suppose that the initial pressure field is $p^{*}$. The intermediate velocities, $u^{*}$ and $w^{*}$, are then computed using the known velocity values at time step *k*. The intermediate velocities’ mathematical formulas, which we obtained from Eqs. (A2) and (A3), the details of which are given in Appendix B, provided in supplementary.

It’s crucial to remember that the continuity requirement might not be satisfied by the intermediate velocities, $u^{*}$ and $w^{*}$. At the following time level, $k+1$, we want to calculate a pressure-correction term, $p'$, and find the associated velocity correction terms, $u'$ and $w'$, in order to guarantee a divergence-free velocity field ($u^{k+1}$ and $w^{k+1}$). The following are the mathematical expressions that may be used to calculate $w'$ and $u'$: Eq. (A2) from Eq. (B1) and Eq. (A3) from Eq. (B2), respectively,

27 $$\begin{aligned} & w' = {\Delta t} \left[ - {\left( \dfrac{\partial p'}{\partial z}\right) ^{k}_{i,lj}} + A_{i,lj}^{k} {\left( \dfrac{\partial p'}{\partial x}\right) ^{k}_{i,lj}} \right] , \end{aligned}$$

28 $$\begin{aligned} & u' = {\Delta t} \left[ - {\dfrac{1}{R} \left( \dfrac{\partial p'}{\partial x}\right) ^{k}_{li,j}} \right] , \end{aligned}$$

where,

29 $$\begin{aligned} & u{\prime } = {u^{k+1}_{li,j}-u^{*}_{li,j}}, \end{aligned}$$

30 $$\begin{aligned} & w{\prime } = {w^{k+1}_{i,lj}-w^{*}_{i,lj}}, \end{aligned}$$

31 $$\begin{aligned} & p{\prime } = {p^{k+1}_{i,j}-p^{*}_{i,j}}. \end{aligned}$$

By the techniques below, we derive a Poisson equation to compute $p'$. After pre-multiplying the latter by *x*, we first differentiate Eq. (27) with respect to *z* and Eq. (28) with respect to *x*. The final expressions, which are shown below without the indices *i*, *j*, *k* for brevity, are

32 $$\begin{aligned} & \dfrac{\partial w'}{\partial z} - \dfrac{x}{R}\dfrac{\partial R}{\partial z} \dfrac{\partial w'}{\partial x} = - \Delta {t} \left[ \dfrac{\partial ^{2} p'}{\partial z^{2}} + \left( \dfrac{x}{R} \dfrac{\partial R}{\partial z}\right) ^{2} \dfrac{\partial ^{2} p'}{\partial x^{2}} + \left( \dfrac{2x}{R^{2}} \left( \dfrac{\partial R}{\partial z}\right) ^{2} - \dfrac{x}{R} \dfrac{\partial ^{2} R}{\partial z^{2}}\right) \dfrac{\partial p'}{\partial x} - \dfrac{2x}{R}\dfrac{\partial R}{\partial z} \dfrac{\partial ^{2} p'}{\partial {z} \partial {x}} \right] , \end{aligned}$$

33 $$\begin{aligned} & \dfrac{\partial (xu')}{\partial x} = - \Delta {t} \left[ \dfrac{x}{R}\dfrac{\partial ^{2} p'}{\partial x^{2}} + \dfrac{1}{R}\dfrac{\partial p'}{\partial x}\right] . \end{aligned}$$

Subsequently, by combining Eq. (32) and Eq. (33) and enforcing the continuity constraint (Eq. (A1)), we derive an equation for pressure correction, which is expressed as

34 $$\begin{aligned} & -\left( \dfrac{\partial w^{*}}{\partial z} - \dfrac{x}{R} \dfrac{\partial R}{\partial z} \dfrac{\partial w^{*}}{\partial x} + \dfrac{1}{xR} \dfrac{\partial (x u^{*})}{\partial x} \right) = \Delta {t} \left[ \left( \dfrac{1}{R^{2}} + \left( \dfrac{x}{R}\dfrac{\partial R}{\partial z} \right) ^{2} \right) \dfrac{\partial ^{2} p'}{\partial x^{2}} \right. \nonumber \\ & \left. + \left( \dfrac{1}{xR^{2}} + \dfrac{2x}{R^{2}} \left( \dfrac{\partial R}{\partial z}\right) ^{2} - \dfrac{x}{R} \dfrac{\partial ^{2} R}{\partial z^{2}} \right) \dfrac{\partial p'}{\partial x} - \dfrac{2x}{R} \dfrac{\partial R}{\partial z} \dfrac{\partial ^{2} p'}{\partial {z} \partial {x}} + \dfrac{\partial ^{2} p'}{\partial z^{2}} \right] . \end{aligned}$$

We solve Eq. (34) to obtain the value of $p'$, applying homogeneous Neumann boundary conditions on all edges and over-relaxing the solution by a factor of 1.3. In order to compute the pressure correction using second-order accurate central difference methods, the equation’s mixed derivative term for $p'$ necessitates the use of a nine-point stencil. By utilizing Eqs. (29)-(31), we can derive the divergence-free velocity field, $u^{k+1}$ and $w^{k+1}$ and along with the corresponding pressure field, $p^{k+1}$. This is achieved by summing up $u'$, $w'$ and $p'$ with $u^{*}$, $w^{*}$, and $p^{*}$, respectively. After obtaining the velocity field, we calculate the Skin Friction or Wall Shear Stress, WSS ($\tau$). The formula^70^ for computing WSS ($\tau$) is expressed as,

35 $$\begin{aligned} & (\tau )_{i,j}^{k} = (\tau _{xz})_{i,j}^{k} \times \cos \left[ \tan ^{-1} \left( \dfrac{\partial R}{\partial z}\right) _{j}^{k} \right] . \end{aligned}$$

Further, to assess the rapid, significant variations with WSS either temporally or spatially, which is critical in the development of atherosclerosis, we study oscillatory shear index (OSI). It is defined based on the temporal changes in WSS values. A measure of how much mobility occurs back and forth along the artery wall in a single heartbeat is called the OSI. Its values range from 0, indicating a consistent WSS direction, to 0.5, signifying a 180$^{\circ }$ change in WSS direction. With *cc* representing the time period of one cardiac cycle, the OSI is determined through the following calculation,

36 $$\begin{aligned} OSI= & \dfrac{1}{2} \left[ 1 - \dfrac{\left| \int ^{cc}_{0} WSS \hspace{1mm} dt\right| }{\int ^{cc}_{0} WSS \hspace{1mm} dt} \right] . \end{aligned}$$

## Numerical results and discussion

### Numerical stability and validation

We begin by conducting a grid convergence test utilizing the Richardson extrapolation method^71^. Extrapolation is carried out in this process based on the results obtained from three distinct grid solutions. We implement three grid refinements in both the radial and axial directions. In the radial direction, we choose the following grid refinements, $\Delta {x}_{1}$ = 0.04, $\Delta {x}_{2}$ = 0.025, and $\Delta {x}_{3}$ = 0.01818. Similarly, in the axial direction, we choose the following grid refinements, $\Delta {z}_{1}$ = 0.072, $\Delta {z}_{2}$ = 0.045, and $\Delta {z}_{3}$ = 0.0329. The grid ratios, $r_{{\gamma }_{1}}$ = 1.6 and $r_{{\gamma }_{2}}$ = 1.388, are roughly the outcome of this. Thus, 25 $\times$ 343, 41 $\times$ 551, and 55 $\times$ 761 are the resultant grids. The average of the refinement ratios is used to compute the grid refinement ratio denoted by $r_{s}$, given by,

37 $$\begin{aligned} r_{s} = \left( \dfrac{r_{{\gamma }_{1}}+r_{{\gamma }_{2}}}{2}\right) = \left( \dfrac{1.6+1.388}{2}\right) = 1.494. \end{aligned}$$

In a grid refinement study, we compute the Grid Convergence Index (GCI), a standardized metric for assessing convergence. The following is the formula for GCI^72^,

38 $$\begin{aligned} GCI = F_{sf} \dfrac{\delta }{r^{p}-1}, \end{aligned}$$

where $F_{{sf}}$ is the safety factor, $\delta$ represents the relative error between two grid conditions in the simulation, and *p*, is the order of convergence defined by,

39 $$\begin{aligned} p = \dfrac{ \ln | \left( f_{{\text {coarse}}} - f_{{\text {medium}}} \right) /\left( f_{{\text {medium}}} - f_{{\text {fine}}} \right) | }{ \ln (r_{s}) }, \end{aligned}$$

where the details of $f_{{coarse}}$, $f_{{medium}}$, and $f_{{fine}}$can be found in^70^. $\delta$ is given by,

$$\begin{aligned} {\delta}_{{\gamma }_{1}} = \dfrac{f_{{\text {coarse}}} - f_{{\text {medium}}}}{f_{\text {medium}}}, \hspace{10mm} {\delta}_{{\gamma }_{2}} = \dfrac{f_{\text {medium}} - f_{\text {fine}}}{f_{\text {fine}}}. \end{aligned}$$

The representative information for calculating the GCI using the previously mentioned formulations is shown in Table 1. In all examples with different shapes, $GCI_{{\gamma }_{2}}$ is found to be less than $GCI_{{\gamma }_{1}}$. A smaller GCI value means that the numerical results are less dependent on the grid size^73^, meaning that more refinement won’t materially change the calculated answers. In order to pick $\Delta {t}$ and ensure a stable solution, we use this information to finalize $\Delta {x}$ and $\Delta {z}$,

**Table 1:** Order of accuracy and GCI.

Shape of stenosis		$\delta _{{\gamma }_{1}}$	$\delta _{{\gamma }_{2}}$	$F_{sf}$	*p*	$GCI_{{\gamma }_{1}}$	$GCI_{{\gamma }_{2}}$
$S_{e}$	$w_{mean}$	0.000506	0.000303	1.25	1.2773	0.000768	0.000728
$S_{h}$	$w_{mean}$	0.032446	0.018728	1.25	1.3217	0.004709	0.043160
$S_{l}$	$w_{mean}$	0.028571	0.016105	1.25	1.3874	0.038838	0.034951
$S_{o}$	$w_{mean}$	0.032025	0.019390	1.25	1.3309	0.004606	0.004430

Following^74^, first, we use *Re* to constrain the time step,

$$\begin{aligned} \Delta {t}_{1} = \text{ min } \left[ \dfrac{Re}{2} \dfrac{{\Delta {z}}^{2} {\Delta {x}}^{2}}{{\Delta {z}}^{2} + {\Delta {x}}^{2}} \right] . \end{aligned}$$

Additionally, another constraint on the time step, derived from the flow field, is provided by

$$\begin{aligned} \Delta {t}_{2} = \text{ min }\left[ \dfrac{{\Delta {z}}}{|w|}, \dfrac{{\Delta {x}}}{|u|} \right] _{i,j}. \end{aligned}$$

For the momentum solver, we therefore choose the following time step $(\Delta {t})$, with $c_{sf}$ $(0< c_{sf} < 1)$ as an additional argument used to provide a seamless computation,

$$\begin{aligned} \Delta {t} = c_{sf} \hspace{0.1cm} \text{ min }\left( \Delta {t}_{1},\Delta {t}_{2}\right) . \end{aligned}$$

**Fig. 2:**
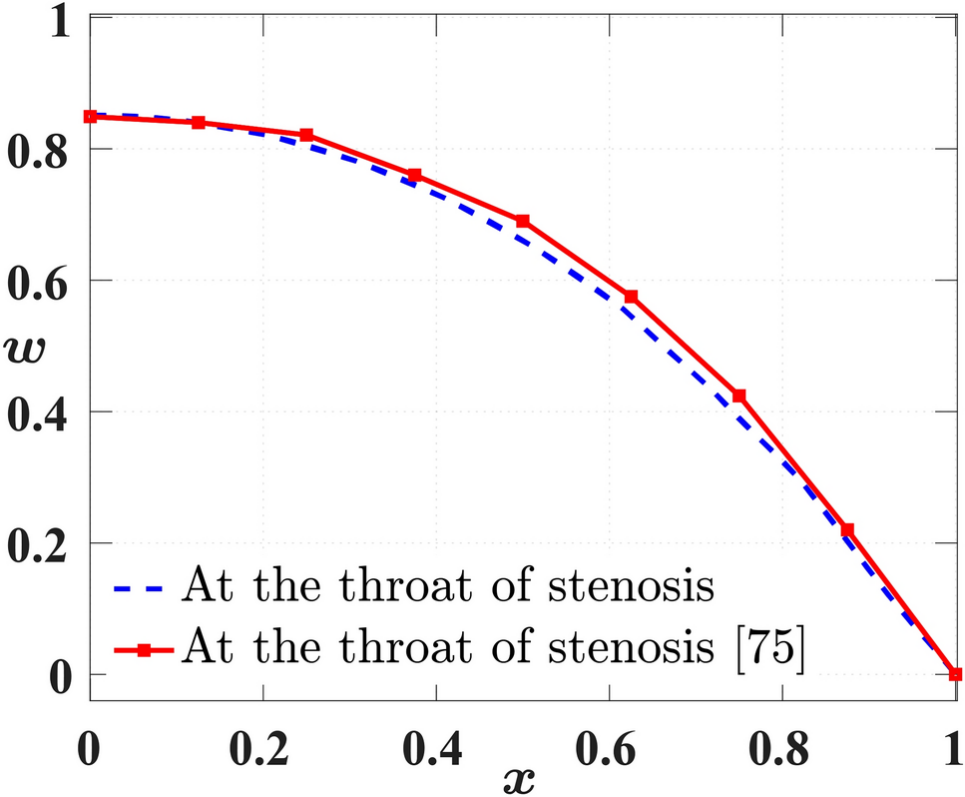
Comparison with the result obtained in the works of Sarifuddin et al.^75^, by fixing, Re = 300, 48% area reduction in the stenosis region.

Finally, we use a benchmark test from the literature^75^ to validate our numerical solver, assuming elliptical-shaped stenosis with a maximum constriction of 48%. We compare the axial velocity profiles along the radial direction at the stenosis throat. In Figure 2, we find that the solution predicted by our solver is in close agreement with the findings of Sarifuddin et al.^75^, who examined blood flow in a stenosed artery with a $48\%$ reduction in area. To compare the results, we represent the approximation obtained by our solver as $w_{ap}$, and that considered from the cited reference as $w_{rf}$. Specifically, we calculate $L_{\infty }$ norm with the formulation, max$|w_{ap}-w_{rf}|$, and further also calculate the maximum percentage in difference between $w_{ap}, w_{rf}$, with the formulation, $\frac {\left( w_{ap}-w_{rf}\right) }{w_{rf}} \times 100$. We found that $L_{\infty }\;\text{ norm }$ is equal to 0.0354 and the maximum percentage difference is $8.352\%$. Our approximation is comparable to two decimal places with the findings of Sarifuddin et al.^75^, with which we proceed further with the parametric studies, presented in detail, together with the results, in the subsection that follows,

### Parametric studies

In our parametric study, while we choose the angle of inclination ($\Theta$) and shape of the stenosis ($S_{j}$, wherein, $j\in \{e,h,l,o\}$) as the geometrical parameters, we choose, flow behavior index (*n*), body acceleration (*G*), frequency of the pulsatile flow at the arterial inlet ($f_{i}$), the viscosity of the fluid ($\mu _{p}$), and average cross-sectional velocity ($u_{0}$) as the flow parameters. Note that the last three flow parameters mentioned are assumed values only at the inlet during the simulation, as they are subjected to vary within the domain influenced by the other flow parameters. We perform our numerical parametric study for a fixed $r_{0}=0.0042$ m and $\rho =1060$ $\text{ kgm}^{-3}$. The parameters, $\mu _{p}$ takes the values, 0.0035 $\text{ kgm}^{-1}\text{ s}^{-1}$, and 0.0055 $\text{ kgm}^{-1}\text{ s}^{-1}$, for normal and high viscosity, respectively; $u_{0} = 0.27$ $\text{ ms}^{-1}$ and $f_{i} = 1.087$ $\text{ s}^{-1}$, for resting condition; $u_{0} = 0.36$ $\text{ ms}^{-1}$ and $f_{i} = 2.381$ $\text{ s}^{-1}$, for exercise condition. The following sections will discuss the remaining parameters and their range case-wise.

#### Influence of geometrical parameters on flow field

**Fig. 3:**
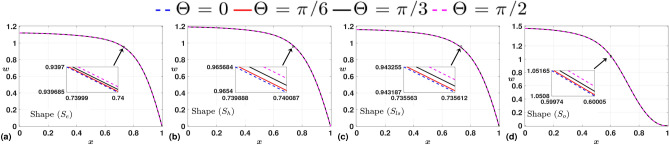
Time-averaged axial velocity (*w*) measured in the radial direction at the throat of stenosis for various angles in each case. We observe a direct proportional relation between the angle and the velocity magnitude.

**Fig. 4:**
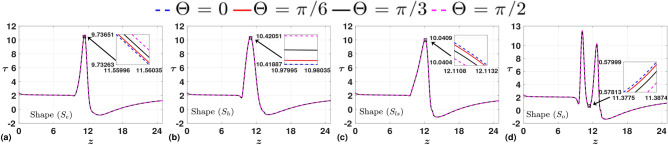
Time-averaged WSS ($\tau$) measured along the arterial wall for various angles in each case. We observe a direct proportional relation between the angle and the WSS peak for $S_{e}$ and $S_{hs}$. The reverse trend is observed for $S_{ls}$ and $S_{o}$.

To understand the effects of the angle of inclination ($\Theta$) and the shape of stenosis ($S_{j}$) on the flow field, we fix the flow parameter, $n=1$, and assume Newtonian flow behavior under normal viscosity ($\mu _{p}=0.0035$ $\text{ kgm}^{-1}\text{ s}^{-1}$) at rest condition ($u_{0}=0.27$ $\text{ ms}^{-1}$, $f_{i}=1.087$ $\text{ s}^{-1}$), resulting in $Re=343$, and $Fr=1.3302$. In Fig. 3, we study the variations in time-averaged axial velocity profile, *w* for all shapes of stenosis, and all four discrete angles, $\Theta$ between 0 to $\frac {\pi }{2}$ with an increment of $\frac {\pi }{6}$. We observe a direct proportional relation between the angle and the velocity magnitude for all shapes of stenosis. This observation correlates with the findings of Awan et al.^19^. However, the order of increment in the velocity magnitude over each case of varying $\Theta$ is considerably less. We observe a similar degree of impact of angles on WSS as depicted in Fig. 4. While we observe a direct proportional relation between the angle and the WSS peak for $S_{e}$ and $S_{h}$, a reverse trend is observed for $S_{l}$. We also observe an inverse relation for the shape $S_{o}$ at the center of the stenotic region. Still, the value does not correlate to its peak and is near zero, corresponding to a near-reverse flow in the domain. Specifically, the negative values of WSS in the post-stenotic region represent reverse flow in the domain. The influence of shape on the flow field is very apparent in the case of shape $S_{o}$, where the time-averaged velocity is greater than that of other shapes. The same trend is observed for WSS, where the maximum of the peaks over varying $\Theta$ is more for the case of shape $S_{o}$. Overall, we infer that for the given case of the Froude number ($Fr=1.3302$), the influence of angle on the flow field is minimal. With that as a lead, we fix the angle of inclination, $\Theta =0$, for the remainder of the article. We now closely investigate shapes’ influence on the flow field at three critical locations in the arterial domain.

**Fig. 5:**
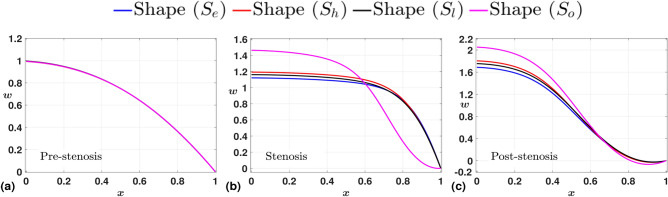
Time-averaged axial velocity (*w*) measured at three different axial locations, (**a**) Pre-stenosis (upstream to stenosis), (**b**) Stenosis (throat of stenosis), (**c**) Post-stenosis (downstream to stenosis). As expected, the velocity almost remains the same before the constriction for all shapes; however, it increases significantly at the throat and post-stenosis for the stenosis shape ($S_{o}$).

**Fig. 6:**
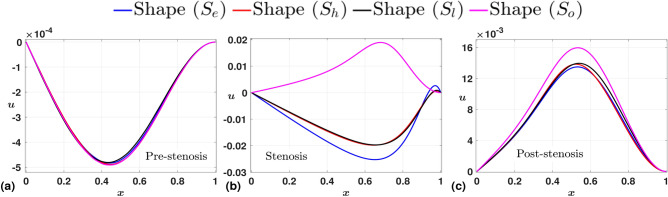
Time-averaged radial velocity (*u*) measured at three different axial locations, (**a**) Pre-stenosis (upstream to stenosis), (**b**) Stenosis (throat of stenosis), (**c**) Post-stenosis (downstream to stenosis). The velocity profiles nearly overlap before the constriction for all shapes; however, for the stenosis shape ($S_{o}$), the profile shoots to a positive value at the center line and remains positive with a higher magnitude downstream of the stenosis.

**Fig. 7:**
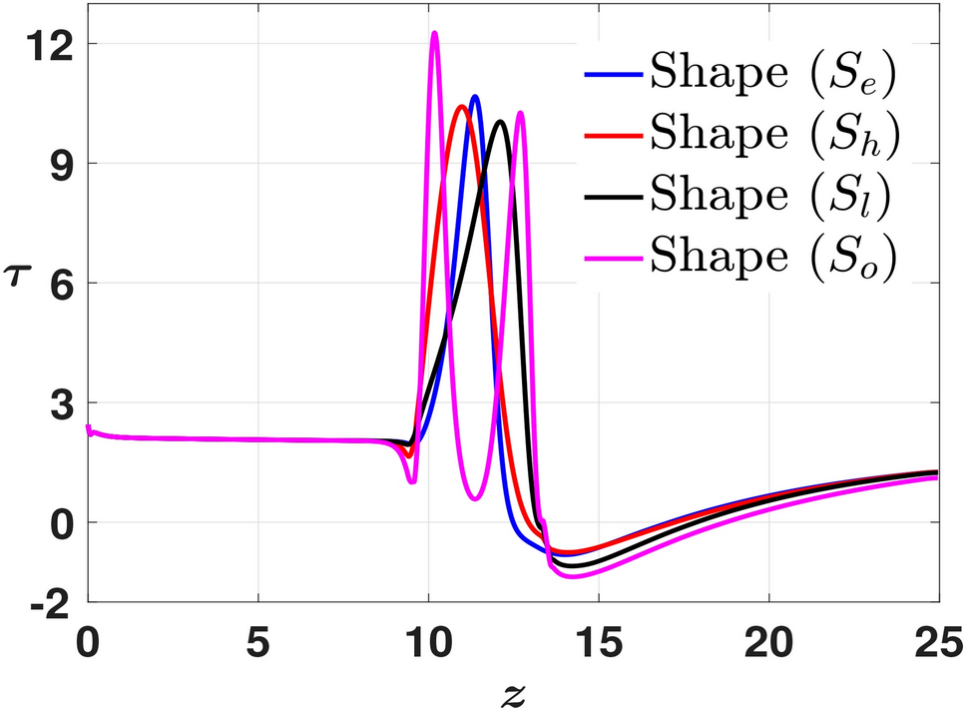
Time-averaged WSS ($\tau$) measured along the arterial wall with the stenosis shapes as the parameter of interest. We observe maximum fluctuations with the maximum peak and drop for stenosis with the shape ($S_{o}$).

Figure 5 shows the variation of time-averaged axial velocity *w* at three different locations. Before constriction, we observe the velocity profiles naturally overlap. However, in the stenotic throat, a clear distinction in the velocity profile appears, the stenosis with the shape $S_{o}$ offering higher velocity. The same is true even in the post-stenotic region, with an overall increase in the magnitude of center-line velocity for all shapes $S_j$. In addition, we observe curved velocity profiles near the wall (Fig. 5(c)), and the negative values signify the reverse flow in the domain, which is an immediate consequence of the constriction. This reverse flow profile is developed early for the shape $S_{o}$, as observed in Fig. 5(b). We understand the collective reasoning in Fig. 6, where we present the variation of time-averaged radial velocity profiles (*u*) at the same locations as for *w*. Again, the profiles nearly overlap in the pre-stenotic region. However, at the stenosis throat, we observe *u* shoots to positive values for the shape $S_{o}$ and remains negative for the other cases. This is because the center-line of the axis for $S_{o}$ corresponds to a region that is an exit point of the first of its two overlapping constrictions, and the velocity vectors are outward because of the relative increase in the area of the cross-section along the domain. This is also the reason for an early onset of reverse flow, as observed in Fig. 5(b). For the same reason, all the radial velocity profiles exhibit positive values at the post-stenosis region downstream of the stenosis (Fig. 5(c)). The reverse flow near the wall in the post-stenosis region is highlighted with the time-averaged WSS plot given in Fig.7, which drops to negative towards the downstream of the stenosis. This drop is more significant for the case with $S_{o}$, which also offers the highest WSS peak. Hence, the fluctuations are clearly maximum for the stenosis with shape $S_{o}$.

**Fig. 8:**
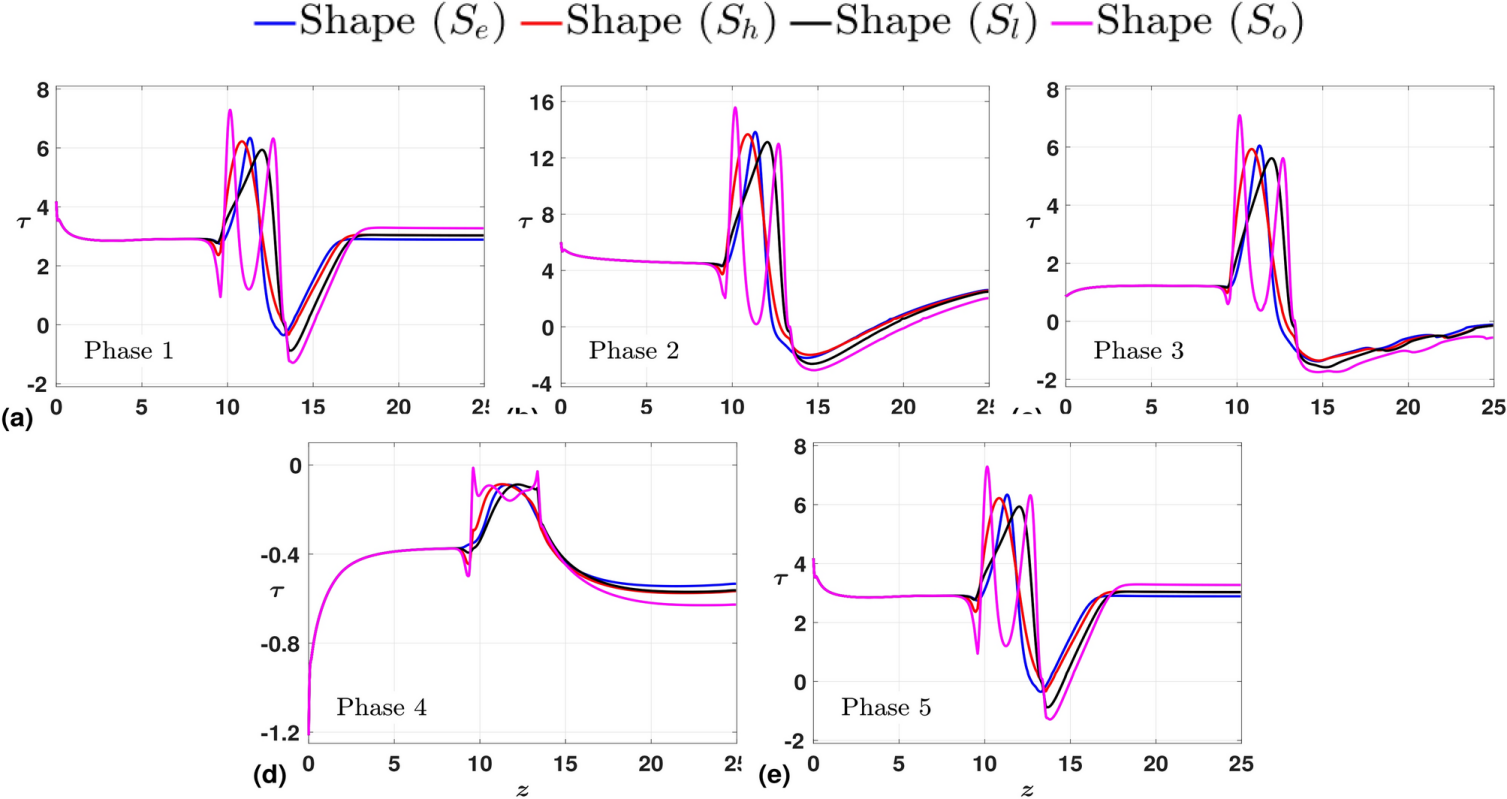
Phase-averaged WSS ($\tau$) measured along the arterial wall with the stenosis shapes as the parameter of interest at (**a**) Phase 1, (**b**) Phase 2, (**c**) Phase 3, (**d**) Phase 4, (**e**) Phase 5.

Finally, to support our inference, we present our findings with the phase-averaged plots. The data points are consolidated for each of the five discrete phases of the inlet pulsatile wave. Then, the average of the same over a certain number (five) of cardiac cycles is considered. We present the phase-averaged WSS plots in Fig. 8. The immediate observation is that the range for the phase averaged $\tau$ is the same for phases $1,3, \text{ and } 5$, and while phases 1 and 5 denote the stationary phases of the inlet pulsatile wave, phase 3 denotes the retardation phase. As a result, phases 2 and 4 relate to the data set that was gathered at the points in the inlet pulsatile wave where the positive half-cycle peak accelerates and the negative half-cycle peak decelerates, respectively. While phases 1 and 5 result in almost similar patterns (phase 5 corresponds to phase 1 for the next cardiac cycle), phase 3 represents the retardation phase, which is a transition of the inlet pulsatile wave from acceleration to deceleration phase. This is the reason we observe in Fig. 8 (c) severe fluctuations in WSS values downstream of the stenosis. At phase 2, we have the maximum flow influx, which results in high WSS peaks. On the contrary, during phase 4, the minimum influx, which is briefly negative, results in negative WSS values throughout the arterial wall. For clarity purposes, the minimum and maximum WSS values for each shape and phase are consolidated in Table 2. Upon analyzing both time-averaged and phase-averaged data, it is evident that the highest flow fluctuations occur for the stenosis with shape $S_{o}$. Therefore, based on this observation, we fix $S_{o}$ as the shape of stenosis to study the influence of flow parameters in the final two subsections.

**Table 2:** Minimum and maximum value of phase-averaged WSS for each phase and shape of the stenosis.

Shape of stenosis	Phase 1		Phase 2		Phase 3		Phase 4		Phase 5	
	min	max	min	max	min	max	min	max	min	max
$S_{e}$	$-0.3527$	6.3400	$-2.2080$	13.840	$-1.3760$	6.0510	$-0.5441$	$-0.0885$	$-0.3507$	6.3400
$S_{h}$	$-0.3369$	6.2230	$-1.9920$	13.670	$-1.3520$	5.9290	$-0.5752$	$-0.0862$	$-0.3351$	6.2190
$S_{l}$	$-0.8821$	5.9370	$-2.6320$	13.110	$-1.5790$	5.6110	$-0.5964$	$-0.0876$	$-0.8728$	5.9290
$S_{o}$	$-1.2900$	7.2760	$-3.0850$	15.580	$-1.7470$	7.0880	$-0.6284$	$-0.0136$	$-1.2890$	7.2880

#### Influence of flow parameters on flow field

**Fig. 9:**
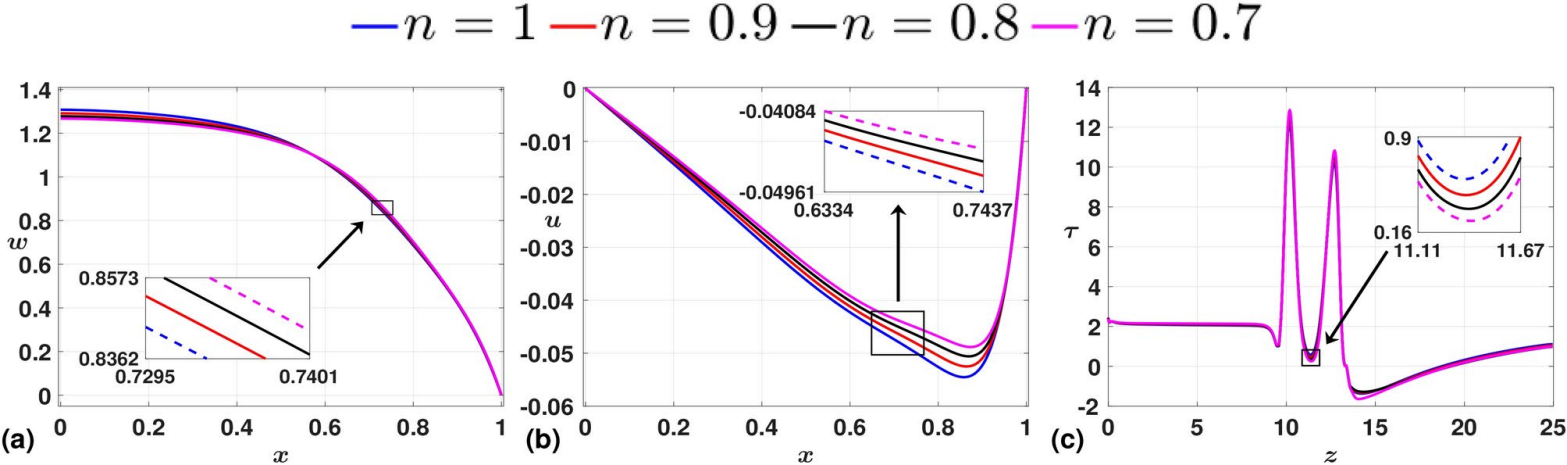
Time-averaged plots for (**a**) axial velocity, *w* (**b**) radial velocity, *u* (**c**) WSS $\tau$. We observe that the non-Newtonian case with $n=0.7$ exhibits a dominant behavior in terms of magnitude in the near wall region.

Following the study on geometric parameters, we now study the influence of flow parameters on the flow field. Initially, we consider the flow behavior index (*n*) as the parameter of interest. In Fig. 9, we present the time-averaged plots, wherein the axial velocity profile (*w*) exhibits a directly proportional relationship between the magnitude of the center-line velocity and the value *n*. However, approaching the wall region, the flow with a lower value of the flow behavior index increases in magnitude, showcasing the dominance of non-Newtonian behavior ($n=0.7$) over that of Newtonian ($n=1$). This phenomenon is more pronounced away from the artery’s axis, where the wall influences the profile shape more than near the axial region ($x=0$). The WSS plot reveals that the degree of reverse flow in the post-stenotic region is more significant for $n=0.7$ than that of its counterparts and that of the Newtonian case with $n=1$. Thus, given the significance of *n* on the flow profile, we consider the flow to be non-Newtonian and fix $n=0.7$ for the following parametric studies.

**Fig. 10:**
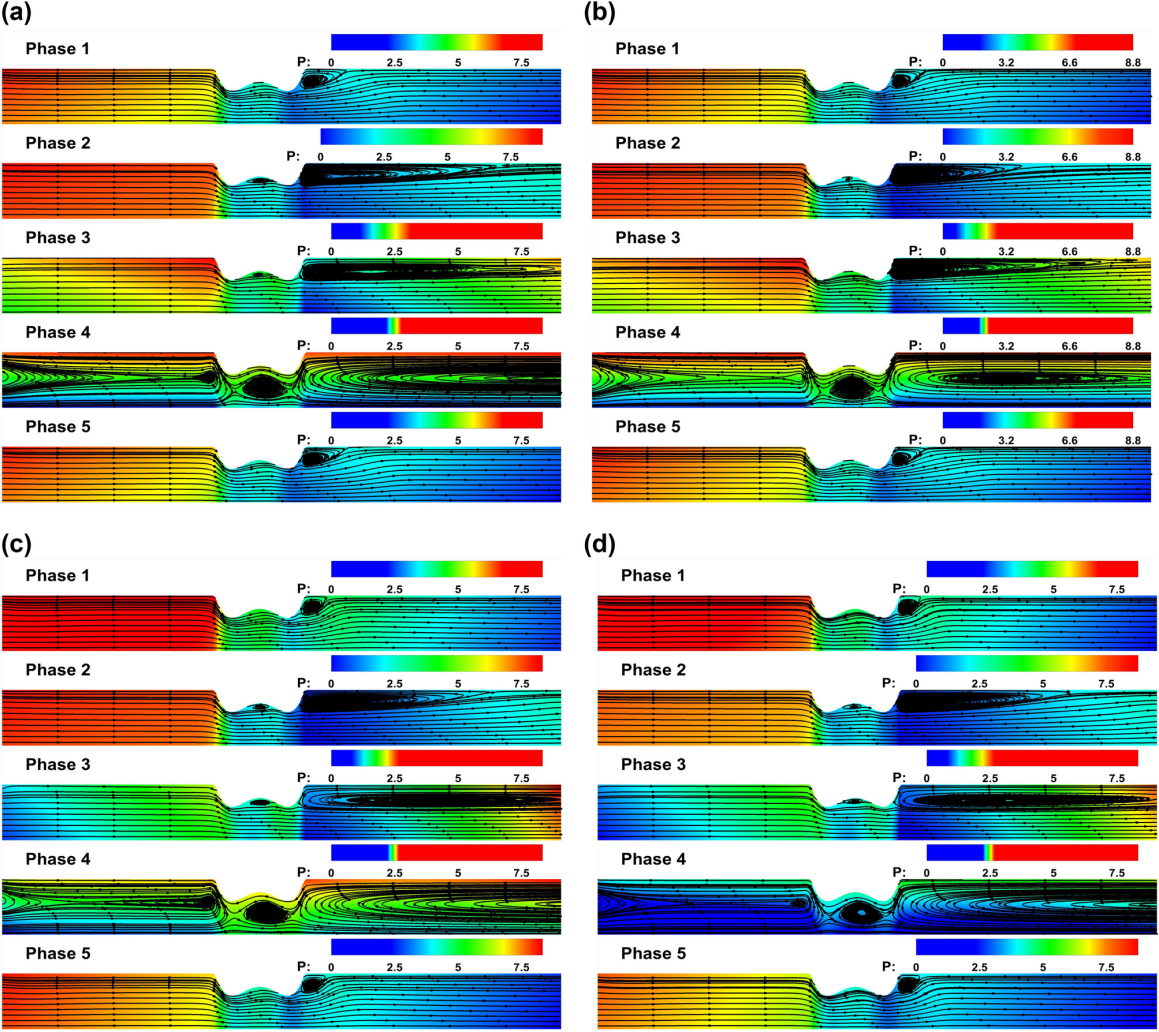
Phase-averaged contour plots for different test cases (**a**) Rest condition with normal viscosity (**b**) Rest condition with high viscosity (first row) (**c**) Exercise condition with normal viscosity (**d**) Exercise condition with high viscosity (second row).

With the non-Newtonian profile for blood flow, we now consider the four critical cases that correlate the viscous nature of blood and the flow conditions that the arterial domain is subjected to, viz., rest or exercise state. The physics of this is decided by the parameters such as average cross-sectional velocity ($u_{0}$), inlet pulsatile frequency $f_{i}$, constant responsible for the onset of acceleration due to gravity, $\Lambda$, and viscosity, $\mu _{p}$. Table 3 provides the complete breakdown of the four possible cases. While *Re* and $W\hspace{-.7mm}o$ depend both on the physiological conditions and viscosity, *Fr* is independent of the viscosity of the fluid. We also observe that the case for the rest condition with hyperviscosity produces the least value for *Re* and $W\hspace{-.7mm}o$, and the case for the exercise condition with normal viscosity has the highest value for *Re* and $W\hspace{-.7mm}o$. In Fig. 10, we plot the phase-averaged velocity field’s contour plot superimposed with the pressure field to understand the resulting variations in the flow field. We analyze phase-wise and emphasize the distinction between the rest and exercise conditions over the different viscosity.

**Table 3:** The complete breakdown of the four cases for varying $u_{0}$, $f_{i}$, $\Lambda$, and $\mu _{p}$ and the resulting *Re*, $W\hspace{-.7mm}o$, *Fr*.

Physiological condition	Parametric values	Normal viscosity	Hyperviscosity
		$\mu _{p}=0.0035$	$\mu _{p}=0.0055$
Rest condition	$u_{0}=0.27$ $f_{i}=1.087$ $\Lambda =0.0$	$Re=343$ $W\hspace{-.7mm}o=6.0404$ $Fr=1.3302$	$Re=219$ $W\hspace{-.7mm}o=4.8186$ $Fr=1.3302$
Exercise condition	$u_{0}=0.36$ $f_{i}=2.780$ $\Lambda =1.0$	$Re=458$ $W\hspace{-.7mm}o=9.6562$ $Fr=1.7735$	$Re=291$ $W\hspace{-.7mm}o=7.7030$ $Fr=1.7735$

**Fig. 11:**
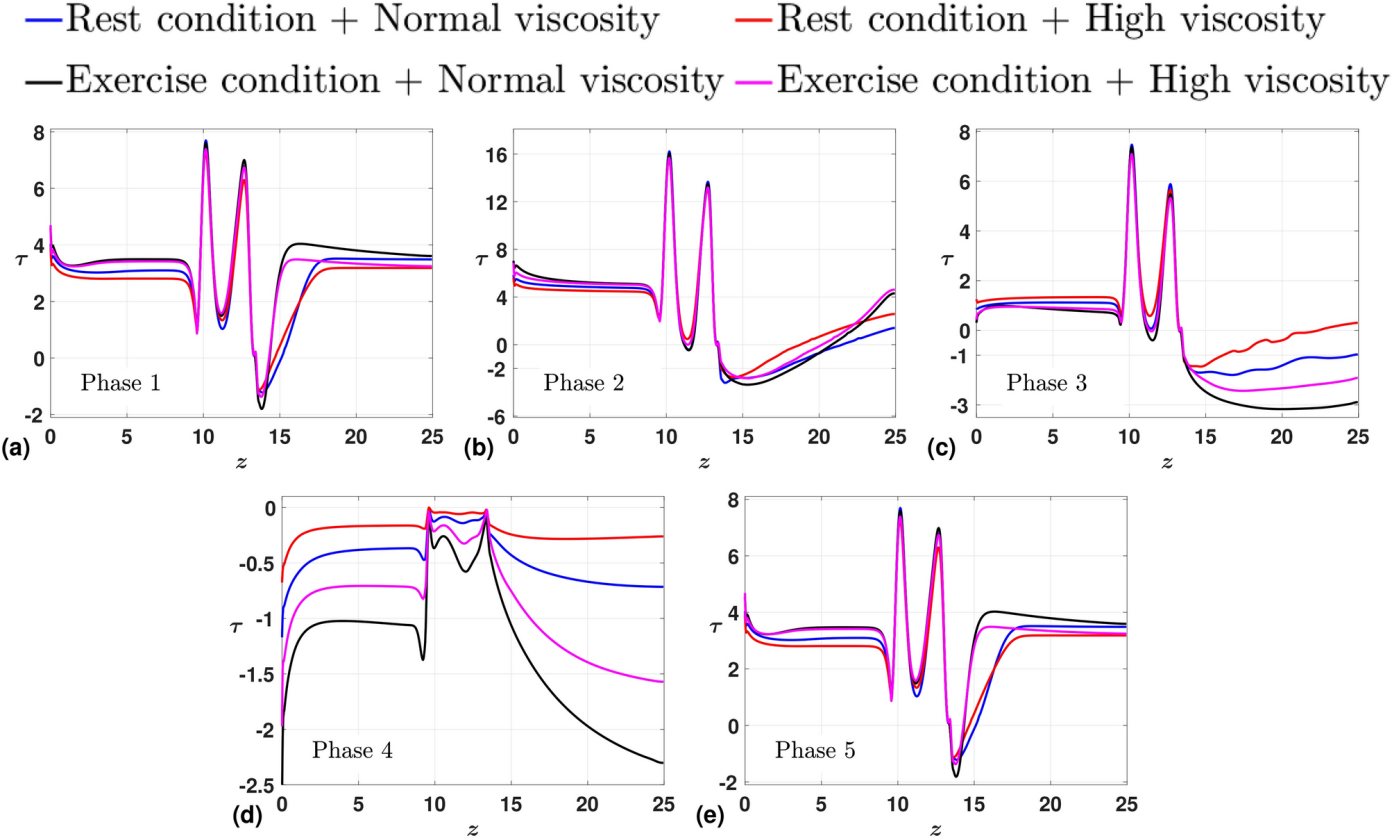
Phase-averaged WSS ($\tau$) measured along the arterial wall with the stenosis shapes as the parameter of interest at (**a**) Phase 1, (**b**) Phase 2, (**c**) Phase 3, (**d**) Phase 4, (**e**) Phase 5.

During the first phase, representative of the beginning of a cardiac cycle, all four plots produce a single recirculation zone near the wall in the post-stenosis region, where there is a significant pressure drop relative to the upstream of the stenosis. In the second phase, as the acceleration phase of the positive half-cycle reaches its peak, the recirculation zone expands in length and size downstream of the stenosis. Notably, cases under exercise conditions exhibit an even higher positive influx (greater $u_{0}$ at the inlet, Table. 3) compared to those under rest conditions. This allows the flow under the exercise condition to regain the positive flow rate near the downstream, thus shortening the recirculation zone. While this may not be apparent with the contour plots, we defer to Fig. 11, where we plot the phase-averaged WSS values for the four considered cases. Specifically, in Fig. 11(b), we can observe that under the exercise conditions, WSS values revert to higher positive values towards the downstream, approaching the outlet. In addition, the recirculation zone is shortened for hyperviscosity cases under rest conditions due to the increased viscosity, which is attributed to lower *Re*, resulting in a more prominent laminar nature in the flow. During the third phase, representative of the retardation phase of the inlet pulsatile wave, it is the onset of the deceleration phase. Under the exercise conditions, we observe an apparent higher pressure field towards the outlet in the near-wall region. This adverse pressure field results in a flow field with an elongated recirculation zone. The same can be inferred in Fig. 11(c), wherein the negative WSS values extend toward the outlet and are more severe under the exercise condition. Physically, this accounts for the substantial local pressure gradient toward the downstream, which culminates in pockets of recirculation zones in the low-pressure regions. These recirculation zones frequently contribute to the accumulation of LDL^59^, ultimately leading to the development of blockages. During the fourth phase, reaching the peak of the deceleration phase of the negative half-cycle, we observe a complete flow reversal in the entirety of the domain. The same is reflected in Fig. 11(d), with the WSS values dropping to negative along the arterial wall. The influence of viscosity is more apparent in this phase, wherein an inverse relation can be drawn between the magnitude of the WSS values and the viscosity. During the fifth phase, the cycle is set to complete, and the flow returns to the initial phase for the start of the next cycle, with all four plots producing a single recirculation zone near the wall in the post-stenosis region. Overall, we observe that under the state exercise condition, the WSS values are more negative toward the downstream, which accounts for more intensified recirculation zones. Recirculation zones contribute to the accumulation of LDL. Under the exercise state, the heart exerts severe pumping, leading to a proportional shoot-up in the severity of the recirculation zones. Clinically, in the long run, this could lead to the complete blockage of the arteries, which can result in cardiac arrest. Thus in the final subsection, we analyze the sequential effect of the rest and exercise conditions on the flow field.

#### Influence of the combined effect of rest and exercise conditions on flow field

**Fig. 12:**
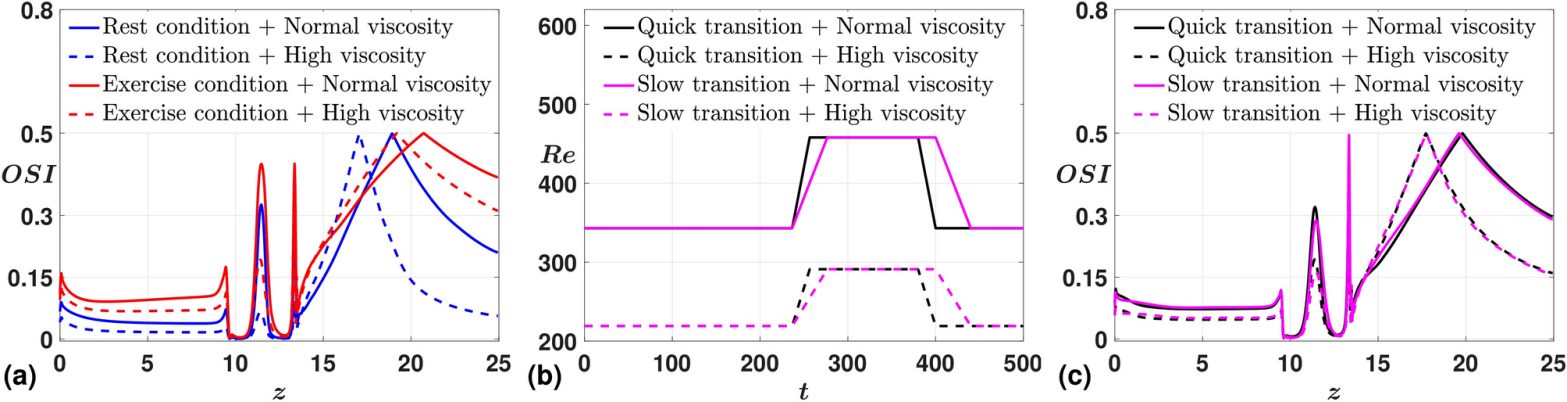
Time-averaged OSI measured along the arterial wall for different case studies. (**a**) Variation of OSI for four stated physiological states, (**b**) Tuning of Reynolds number over time, (**c**) Variation of OSI for different transition rates.

In the final discussion, we consider test cases with the combined influence of resting and exercise conditions on the flow field. We model the cases by considering the sequential effect, wherein in the first four cycles, the domain is in the resting state, followed by a transition onto the exercise state for the following four cycles and eventually transitioning back to the resting state. We also consider the transition rate as a critical parameter of interest and conduct the study for two cases (in terms of viscosity) at two different rates. As an illustrative representation, in Fig. 12 (b), we present the variation of *Re* for two different rates. In Fig. 12 (a) and (c), we exhibit the time-averaged OSI values for the cases considered in the previous subsection, summarised in (Table.3), alongside the combined effect of those cases at two different transition rates. The significance of considering OSI is that the near-wall regions, prone to the progression of atherosclerosis, are distinguished by a characterized diminished WSS that undergoes directional fluctuations. This is associated with an elevated OSI, which is mathematically formulated as (Eq. (36)). In Fig. 12 (a), we observe a consolidated version of our previous discussion, wherein the case in which the domain under the exercise state results in a more intensified recirculation zone. In Fig. 12 (c), we observe that the cases with quick transition, for each considered case of the viscosity, resulted in slightly higher OSI values around the stenotic region. The higher value in OSI correlates to an intensified recirculation zone within the domain in proximity to the arterial constriction. This illustrates the possible impact of a sudden transition from a resting state to an exercise or an active state, which can lead to a significant unfavorable reverse flow in the arterial domain.

## Conclusion

Atherosclerosis is one of the leading causes of death worldwide. Long-term dangers are significant when undiagnosed persistent stenosis increases heart strain. In order to simulate non-Newtonian blood flow in arterial stenosis, this work offers a numerical solver based on pressure correction in two dimensions. The solution incorporates an orthogonal coordinate transformation and is based on a modified version of the SIMPLE method in primitive variables. After validating the solver, we conduct a parametric study to explore how geometric and flow parameters affect the flow field. The parameters used are representative of real physiological conditions. The key findings of this study include:

1. With a minimal variation, we observe that increasing the angle of inclination ($\Theta$) leads to an increase in the magnitude of the center-line velocity.
2. Amongst the different shapes considered, overlapping-shaped stenosis significantly impacts the flow field, with a higher fluctuations in phase-averaged WSS plots.
3. While near the axial region, the Newtonian effect dominates, an elevated velocity is evident in the near-wall region in the non-Newtonian case. This highlights the significance of considering these factors in determining the flow profile.
4. In the analysis of both resting and exercise states, we note that during the exercise condition, the WSS values indicate more intensified recirculation zones, specifically during the deceleration phase of the negative half-cycle of the inlet pulsatile wave.
5. Finally, transitioning from a resting state to an exercise state, the findings indicate that cases with a rapid transition for each viscosity scenario examined exhibit slightly elevated OSI values in the vicinity of the stenotic region. The increased OSI values are associated with the undesirable progression of plaque development along the arterial wall.

The analysis conducted in this study concerning flow patterns and recirculation zones could be applied to arteries of clinical significance characterized by lower Reynolds numbers and their susceptibility to the progression of stenosis. In addition to this, the inclusion of instantaneous data for Womersley solution flow, and the resulting phase-shift^76^ between pressure wave and WSS is worth investigating and is considered as a part of the immediate-future work.

## Supplementary Information

Supplementary Information.

## Data Availability

The datasets generated and/or analysed during the current study are not publicly available due to it (raw data) being incorporated as a base code for its further development but are available from the corresponding author on reasonable request.
