# Supplementary material for: Study of blood flow patterns in a stenosed artery through the combined effect of body acceleration and generalized womersley solution
Source: Sci Rep. 2025 Jan 13;15:1845. doi: 10.1038/s41598-025-85566-2 (PMC11729896; doi:10.1038/s41598-025-85566-2)
Supplement: Supplementary file 1 — Supplementary Information. [file 41598_2025_85566_MOESM1_ESM.pdf]

Manuscript Title: Study of Blood Flow Patterns in a Stenosed Artery Through the Combined Effect of Body Acceleration and Generalized Womersley Solution

Manuscript ID: 809f50df-6c75-47b9-a208-6496bf64b0d0 v1.0

October 11, 2024

A Appendix A

Discrete form of the governing Eqs. (19)-(21),

$$\left(\frac{\partial(xu)}{\partial x}\right)_{i,j}^{k+1}+\left(xR\frac{\partial w}{\partial z}\right)_{i,j}^{k+1}-\left(x^2\frac{\partial w}{\partial x}\right)_{i,j}^{k+1}\left(\frac{\partial R}{\partial z}\right)_{j}^{k+1}=0, \tag{A1}$$

$$\begin{aligned}\frac{w_{i,lj}^{k+1}-w_{i,lj}^{k}}{\Delta t}&=\left(\frac{x}{R}\frac{\partial R}{\partial t}\right)_{i,lj}^{k}\left(\frac{\partial w}{\partial x}\right)_{i,lj}^{k}-\left(\frac{1}{R}\frac{\partial(uw)}{\partial x}\right)_{i,lj}^{k}-\left(\frac{\partial w^2}{\partial z}\right)_{i,lj}^{k}\\&+A_{i,j}^{k}\left(\frac{\partial w^2}{\partial x}\right)_{i,lj}^{k}-\left(\frac{uw}{xR}\right)_{i,lj}^{k}-\left(\frac{\partial p}{\partial z}\right)_{i,lj}^{k+1}+A_{i,lj}^{k}\left(\frac{\partial p}{\partial x}\right)_{i,lj}^{k+1}\\&+\frac{1}{Re}\left[\frac{\gamma}{xR}\left\{\frac{\partial u}{\partial z}-A\frac{\partial u}{\partial x}+\frac{1}{R}\frac{\partial w}{\partial x}\right\}+\left(\frac{\gamma}{R}\left\{\frac{\partial^2 u}{\partial z\partial x}-A\frac{\partial^2 u}{\partial x^2}-\frac{1}{R}\frac{\partial R}{\partial z}\frac{\partial u}{\partial x}+\frac{1}{R}\frac{\partial^2 w}{\partial x^2}\right\}\right)\right.\\&+\frac{1}{R}\left(\left\{\frac{\partial u}{\partial z}-A\frac{\partial u}{\partial x}+\frac{1}{R}\frac{\partial w}{\partial x}\right\}\frac{\partial\gamma}{\partial x}\right)\\&\left.+2\gamma\left(\frac{\partial^2 w}{\partial z^2}-2A\frac{\partial^2 w}{\partial z\partial x}-\frac{x}{R}\frac{\partial^2 R}{\partial z^2}\frac{\partial w}{\partial x}+A^2\frac{\partial^2 w}{\partial x^2}\right)+2\left(\frac{\partial w}{\partial z}-A\frac{\partial w}{\partial x}\right)\left(\frac{\partial\gamma}{\partial z}-A\frac{\partial\gamma}{\partial x}\right)\right]_{i,lj}^{k}\\&+\frac{\sin\Theta}{Fr^2}+\frac{4\Lambda}{Re}\cos(\omega_b t^2+\phi),\end{aligned} \tag{A2}$$

$$\begin{aligned}\frac{u_{li,j}^{k+1}-u_{li,j}^{k}}{\Delta t}&=\left(\frac{x}{R}\frac{\partial R}{\partial t}\right)_{li,j}^{k}\left(\frac{\partial u}{\partial x}\right)_{li,j}^{k}-\left(\frac{1}{R}\frac{\partial(u^2)}{\partial x}\right)_{li,j}^{k}-\left(\frac{\partial uw}{\partial z}\right)_{li,j}^{k}\\&+A_{li,j}^{k}\left(\frac{\partial uw}{\partial x}\right)_{li,j}^{k}-\left(\frac{u^2}{xR}\right)_{li,j}^{k}-\left(\frac{1}{R}\frac{\partial p}{\partial x}\right)_{li,j}^{k+1}\\&+\frac{1}{Re}\left[\left(\frac{2\gamma}{xR^2}\frac{\partial u}{\partial x}\right)+\frac{2\gamma}{R^2}\frac{\partial^2 u}{\partial x^2}+\frac{2}{R^2}\frac{\partial u}{\partial x}\frac{\partial\gamma}{\partial x}\right.\\&+\gamma\left(\frac{\partial^2 u}{\partial z^2}-A\frac{\partial^2 u}{\partial z\partial x}+\frac{x}{R^2}\left(\frac{\partial R}{\partial z}\right)\frac{\partial u}{\partial x}-\frac{x}{R}\frac{\partial^2 R}{\partial z^2}\frac{\partial u}{\partial x}+\frac{1}{R}\frac{\partial^2 w}{\partial z\partial x}-\frac{1}{R^2}\frac{\partial R}{\partial z}\frac{\partial w}{\partial x}\right)\\&\left.+\gamma A\left(\frac{\partial^2 u}{\partial z\partial x}-A\frac{\partial^2 u}{\partial x^2}-\frac{1}{R}\frac{\partial R}{\partial z}\frac{\partial u}{\partial x}+\frac{1}{R}\frac{\partial^2 w}{\partial x^2}\right)+\left(\frac{\partial u}{\partial z}-A\frac{\partial u}{\partial x}+\frac{1}{R}\frac{\partial w}{\partial x}\right)\left(\frac{\partial\gamma}{\partial z}-A\frac{\partial\gamma}{\partial x}\right)\right]_{li,j}^{k}\\&+\frac{\cos\Theta}{Fr^2}.\end{aligned} \tag{A3}$$

B Appendix B

Mathematical expressions for intermediate velocities, from Eqs. (A2) and (A3),

$$
\begin{aligned}
\frac{w^*_{i,lj} - w^k_{i,lj}}{\Delta t} =& \left(\frac{x}{R}\frac{\partial R}{\partial t}\right)^k_{i,lj} \left(\frac{\partial w}{\partial x}\right)^k_{i,lj} - \left(\frac{1}{R}\frac{\partial (uw)}{\partial x}\right)^k_{i,lj} - \left(\frac{\partial w^2}{\partial z}\right)^k_{i,lj} \\
&+A^k_{i,j}\left(\frac{\partial w^2}{\partial x}\right)^k_{i,lj} - \left(\frac{uw}{xR}\right)^k_{i,lj} - \left(\frac{\partial p}{\partial z}\right)^*_{i,lj} + A^k_{i,lj}\left(\frac{\partial p}{\partial x}\right)^*_{i,lj} \\
&+\frac{1}{Re}\left[\frac{\gamma}{xR}\left\{\frac{\partial u}{\partial z} - A\frac{\partial u}{\partial x} + \frac{1}{R}\frac{\partial w}{\partial x}\right\} + \left(\frac{\gamma}{R}\left\{\frac{\partial^2 u}{\partial z \partial x} - A\frac{\partial^2 u}{\partial x^2} - \frac{1}{R}\frac{\partial R}{\partial z}\frac{\partial u}{\partial x} + \frac{1}{R}\frac{\partial^2 w}{\partial x^2}\right\}\right)\right. \\
&+\frac{1}{R}\left(\left\{\frac{\partial u}{\partial z} - A\frac{\partial u}{\partial x} + \frac{1}{R}\frac{\partial w}{\partial x}\right\}\frac{\partial \gamma}{\partial x}\right) \\
&\left.+2\gamma\left(\frac{\partial^2 w}{\partial z^2} - 2A\frac{\partial^2 w}{\partial z \partial x} - \frac{x}{R}\frac{\partial^2 R}{\partial z^2}\frac{\partial w}{\partial x} + A^2\frac{\partial^2 w}{\partial x^2}\right) + 2\left(\frac{\partial w}{\partial z} - A\frac{\partial w}{\partial x}\right)\left(\frac{\partial \gamma}{\partial z} - A\frac{\partial \gamma}{\partial x}\right)\right]^k_{i,lj} \\
&+\frac{\sin\Theta}{Fr^2} + \frac{4\Lambda}{Re}\cos(\omega_b t^k + \phi), \qquad \text{(B1)}
\end{aligned}
$$

$$
\begin{aligned}
\frac{u^*_{li,j} - u^k_{li,j}}{\Delta t} =& \left(\frac{x}{R}\frac{\partial R}{\partial t}\right)^k_{li,j} \left(\frac{\partial u}{\partial x}\right)^k_{li,j} - \left(\frac{1}{R}\frac{\partial (u^2)}{\partial x}\right)^k_{li,j} - \left(\frac{\partial uw}{\partial z}\right)^k_{li,j} \\
&+A^k_{li,j}\left(\frac{\partial uw}{\partial x}\right)^k_{li,j} - \left(\frac{u^2}{xR}\right)^k_{li,j} - \left(\frac{1}{R}\frac{\partial p}{\partial x}\right)^*_{li,j} \\
&+\frac{1}{Re}\left[\left(\frac{2\gamma}{xR^2}\frac{\partial u}{\partial x}\right) + \frac{2\gamma}{R^2}\frac{\partial^2 u}{\partial x^2} + \frac{2}{R^2}\frac{\partial u}{\partial x}\frac{\partial \gamma}{\partial x}\right. \\
&+\gamma\left(\frac{\partial^2 u}{\partial z^2} - A\frac{\partial^2 u}{\partial z \partial x} + \frac{x}{R^2}\left(\frac{\partial R}{\partial z}\right)\frac{\partial u}{\partial x} - \frac{x}{R}\frac{\partial^2 R}{\partial z^2}\frac{\partial u}{\partial x} + \frac{1}{R}\frac{\partial^2 w}{\partial z \partial x} - \frac{1}{R^2}\frac{\partial R}{\partial z}\frac{\partial w}{\partial x}\right) \\
&\left.+\gamma A\left(\frac{\partial^2 u}{\partial z \partial x} - A\frac{\partial^2 u}{\partial x^2} - \frac{1}{R}\frac{\partial R}{\partial z}\frac{\partial u}{\partial x} + \frac{1}{R}\frac{\partial^2 w}{\partial x^2}\right) + \left(\frac{\partial u}{\partial z} - A\frac{\partial u}{\partial x} + \frac{1}{R}\frac{\partial w}{\partial x}\right)\left(\frac{\partial \gamma}{\partial z} - A\frac{\partial \gamma}{\partial x}\right)\right]^k_{li,j} \\
&+\frac{\cos\Theta}{Fr^2}. \qquad \text{(B2)}
\end{aligned}
$$
